# Supplementary material for: Systematic approach to school-based assessments for autism spectrum disorders to reduce inequalities: a feasibility study in 10 primary schools
Source: BMJ Open. 2021 Jan 17;11(1):e041960. doi: 10.1136/bmjopen-2020-041960 (PMC7813406; doi:10.1136/bmjopen-2020-041960)
Supplement: Author's manuscript [file bmjopen-2020-041960.draft_revisions.pdf]

BMJ Open

BMJ Open is committed to open peer review. As part of this commitment we make the peer review history of every article we publish publicly available.

When an article is published we post the peer reviewers' comments and the authors' responses online. We also post the versions of the paper that were used during peer review. These are the versions that the peer review comments apply to.

The versions of the paper that follow are the versions that were submitted during the peer review process. They are not the versions of record or the final published versions. They should not be cited or distributed as the published version of this manuscript.

BMJ Open is an open access journal and the full, final, typeset and author-corrected version of record of the manuscript is available on our site with no access controls, subscription charges or pay-per-view fees (http://bmjopen.bmj.com).

If you have any questions on BMJ Open's open peer review process please email info.bmjopen@bmj.com

BMJ Open

A systematic approach to school based assessments for Autism Spectrum Disorders to reduce inequalities? A feasibility study in ten primary schools.

Journal:	*BMJ Open*
Manuscript ID	bmjopen-2020-041960
Article Type:	Original research
Date Submitted by the Author:	25-Jun-2020
Complete List of Authors:	Wright, Barry; Hull York Medical School University of York<br>Konstantopoulou, Kalliopi; Leeds Community Healthcare NHS Trust, Child and Adolescent Psychiatry<br>Sohal, Kuldeep; Bradford Institute for Health Research<br>Kelly, Brian; Bradford Institute for Health Research<br>Morgan, Geoff; Educational Psychology Team, City of Bradford, Metropolitan District Council<br>Hulin, Cathy; Bradford Institute for Health Research<br>Mansoor, Sara; Bradford District Care NHS Foundation Trust<br>Mon-Williams, Mark; University of Leeds Department of Psychology
Keywords:	Community child health < PAEDIATRICS, Child & adolescent psychiatry < PSYCHIATRY, Developmental neurology & neurodisability < PAEDIATRICS

SCHOLARONE™
Manuscripts

I, the Submitting Author has the right to grant and does grant on behalf of all authors of the Work (as defined in the below author licence), an exclusive licence and/or a non-exclusive licence for contributions from authors who are: i) UK Crown employees; ii) where BMJ has agreed a CC-BY licence shall apply, and/or iii) in accordance with the terms applicable for US Federal Government officers or employees acting as part of their official duties; on a worldwide, perpetual, irrevocable, royalty-free basis to BMJ Publishing Group Ltd ("BMJ") its licensees and where the relevant Journal is co-owned by BMJ to the co-owners of the Journal, to publish the Work in this journal and any other BMJ products and to exploit all rights, as set out in our licence.

The Submitting Author accepts and understands that any supply made under these terms is made by BMJ to the Submitting Author unless you are acting as an employee on behalf of your employer or a postgraduate student of an affiliated institution which is paying any applicable article publishing charge ("APC") for Open Access articles. Where the Submitting Author wishes to make the Work available on an Open Access basis (and intends to pay the relevant APC), the terms of reuse of such Open Access shall be governed by a Creative Commons licence – details of these licences and which Creative Commons licence will apply to this Work are set out in our licence referred to above.

Other than as permitted in any relevant BMJ Author's Self Archiving Policies, I confirm this Work has not been accepted for publication elsewhere, is not being considered for publication elsewhere and does not duplicate material already published. I confirm all authors consent to publication of this Work and authorise the granting of this licence.

For peer review only

V6 18. 06. 2020

A systematic approach to school based assessments for Autism Spectrum Disorders to reduce inequalities? A feasibility study in ten primary schools.

Professor Barry Wright[1]
Dr Konstantopoulou Kalliopi[2]
Kuldeep Sohal [3]
Dr Brian Kelly[3]
Dr Geoff Morgan [4]
Cathy Hulin[3]
Dr Sara Mansoor[5]
Professor Mark Mon-Williams[6]

1 Hull York Medical School, University of York

2 Leeds Community Healthcare NHS Foundation Trust

3 Bradford Institute for Health Research

4 Educational Psychology Team, City of Bradford, Metropolitan District Council

5 Bradford District Care NHS Foundation Trust

6 Department of Psychology, University of Leeds.

Corresponding author email address: artekon84@gmail.com

For peer review only

Abstract:

Objectives: This was a pilot study to explore whether the Early Years Foundation Stage Profile (EYFSP) carried out by teachers at the end of Reception year, followed by the Social Communication Questionnaire (SCQ) can lead to an earlier identification of children with Autism Spectrum Disorders (ASD), earlier access to intervention and reduce inequity in access to assessment and intervention.

Design: Pragmatic prospective cohort

Setting: 10 primary schools from the SHINE project in Bradford

Participants: 587 from 10 schools who transitioned from Reception to Year 1 in July 2017 and had the EYFSP completed were finally included in the study

For peer review only - http://bmjopen.bmj.com/site/about/guidelines.xhtml

V6 18. 06. 2020

Interventions: The assessment involved a team of three multidisciplinary staff who completed the Autism Diagnostic Interview Revised (ADI-R), the Autism Diagnostic Observation Schedule Version2 (ADOS-2), classroom observations with an ASD checklist, a teacher based ASD questionnaire and a final consensus meeting.

Primary outcome measure: NICE guideline compliant clinical diagnosis of ASD.

Secondary outcome measures: age of diagnosis, demographic data and feasibility parameters.

Results: Children who scored low on the EYFS were more likely to score above the SCQ threshold of 12(indicating potential autism), 50% compared to 19% of children not scoring low on the EYFS ($p < 0.001$). All children scoring above the SCQ received a full autism assessment; children who scored low on the EYFS were more likely to be diagnosed with autism (and other developmental issues) compared to those who did not score low on the EYFS.

Conclusions: We identified 9 new children with a diagnosis of ASD, all from ethnic minorities suggesting that this process may be addressing inequalities in early diagnosis found in previous studies. All children who scored above the threshold in the SCQ, required support and this was because the EYFSP questionnaire preceded it thereby including at risk children.

Strengths and limitations of the study:

- Consent was sought from all parents regardless of language by flexible use of interpreters.
- Education and Health data was shared yielding significant benefits
- We applied the SCQ (cut off of 12) to the children who scored 9 and below in the EYFSP and a 15% random sub-sample of children from the high EYFSP group (above 10)
- All children with a score of 12 or above on the SCQ received a detailed comprehensive ASD assessment and the rest had a teachers' screening questionnaire
- Any child who had already had a diagnosis on the Autism Spectrum from the local diagnostic services was also noted

Introduction

What is autism

Autism Spectrum Disorders (ASD) occur in approximately 1.6% of the UK population (1).

ASD is a neurodevelopmental condition that often includes a range of repetitive behaviours,

For peer review only

V6 18. 06. 2020

preoccupations and interests (2), and large differences in social communication development from neuro-typically developing individuals (3). This leads to a need for different approaches to education (4) and parenting (5); (6) which can be costly for local authorities (7) and stressful for parents and family (8); (9).

Early identification

Early identification and Early intervention has shown initial promise in improving outcomes (10); (5). Whilst screening young children in early education settings has been attempted it identifies large numbers of children (14%) with relatively low numbers identified with ASD (11) making cost effective whole population screening problematic (12), More nuanced approaches need to be developed. One promising approach would be to identify at risk populations and use screening and assessment processes within those groups (12). How to identify risk populations requires further research.

Early Years Foundation Stage Profile (EYFSP)

A large survey of parents in the UK describes late diagnosis in primary school despite symptoms being present from infancy (13) and the Care Quality Commission found children with ASD having long waits for diagnosis and interventions (14). Recent studies suggest that using the Early Years Foundation Stage Profile (15) may identify children with higher risk of having an ASD(16). The EYFSP is completed by teachers in England at the end of the reception year and scores 17 different domains of development in terms of whether a child is at an expected level, ahead or behind that level (15). It is used as a mechanism for flagging children who may need additional help in school and to benchmark UK school profiles.

Equality of Access

Recent work by the same group has also shown that the diagnosis of autism is less likely to be made early in families from poor backgrounds or from families from ethnic minority groups (17) showing inequalities reported elsewhere (18). This problem with equity of

For peer review only - http://bmjopen.bmj.com/site/about/guidelines.xhtml

V6 18. 06. 2020

access would be well served by having a more widely available process for identifying children for neurodevelopmental disorder assessment as early as is practicable. One mechanism for improving equity of access is school based assessment (19).

Reasons for feasibility work

To plan a larger study it is necessary to gather feasibility information for improved assessment processes. We report a feasibility study of a two stage screening process involving the EYFSP followed by an established well validated ASD screening questionnaire, the Social Communication Questionnaire (SCQ) (20). We sought to test the feasibility of a process where children went through this screening process and were then assessed more comprehensively for ASD in schools with education and health professionals working together over one day.

Methodology

Background

This research was set within the larger Born in Bradford cohort research (21). We obtained consent from 10 primary schools in an existing project, the SHINE project. The SHINE group is a consortium of ten primary schools that act as a testbed for new approaches to improve services, reduce inequalities and test innovation (22). We obtained ethical approval from University of Leeds and Bradford Teaching Hospitals NHS Foundation Trust (IRAS Number: 233328).

Consent

All parents were approached with a family information leaflet and a consent form. A researcher was available by phone, email or face to face for those wishing to discuss this further. Interpreters were available because many of the population had a first language that was not English.

Design

For peer review only - http://bmjopen.bmj.com/site/about/guidelines.xhtml

V6 18. 06. 2020

596 children in year 5 were available in 10 primary schools and we approached all of those who had received an Early Years Foundation Stage Profile scored by their teachers at the end of reception year in the summer of 2017.

The study was not powered to look for differences but designed to test feasibility for a larger study.

Measures

A screening measure to identify children at risk was derived from five items of the EYFSP carried out by teachers at the end of reception year from the four main symptom areas defined in the research diagnostic criteria for ASD namely social reciprocity, language and communication, imagination delays and repetitive and stereotyped patterns of behaviour. This is described in more detail in a previous study (16). We chose a score cut off of 9 which a previous study found to be statistically significantly associated with over 50 times the risk of autism, compared to children not scoring low on the EYFSP sub-item score: 52.7 (95% CI: 25.2 - 110.5). (16). Children were dichotomously groped into 'low' (9 or below) and 'high' (10 or above).

The teachers of children with low EYFSP scores and a 15% randomised sub-group of those with high scores (10 or more) completed a Social Communication Questionnaire (SCQ) (23), which is a well-established validated autism screening questionnaire with good sensitivity and specificity scores. In previous studies the SCQ has been found to be helpful with young children in identifying ASD (24). A threshold score of 12 or above on the SCQ was chosen, based on previous research (25); (26) suggesting this is the best cut off for the optimum sensitivity to discriminate between children with and without ASD.

Methods

Data linkage allowed us to combine school and health data (26).

For peer review only - http://bmjopen.bmj.com/site/about/guidelines.xhtml

V6 18. 06. 2020

All those children and families with low EYFSP scores and above threshold SCQ (>12) were offered a NICE guideline compliant ASD Assessment, with additional clinical screening assessment for other developmental problems. A 15% randomised sub-group of those scoring high (10 or more) in EYFSP had the SCQ completed and those who scored high (10 or more) in EYFSP and 12 or above on the SCQ were then also assessed comprehensively in the same way (see figure 1).

Figure 1: Study design

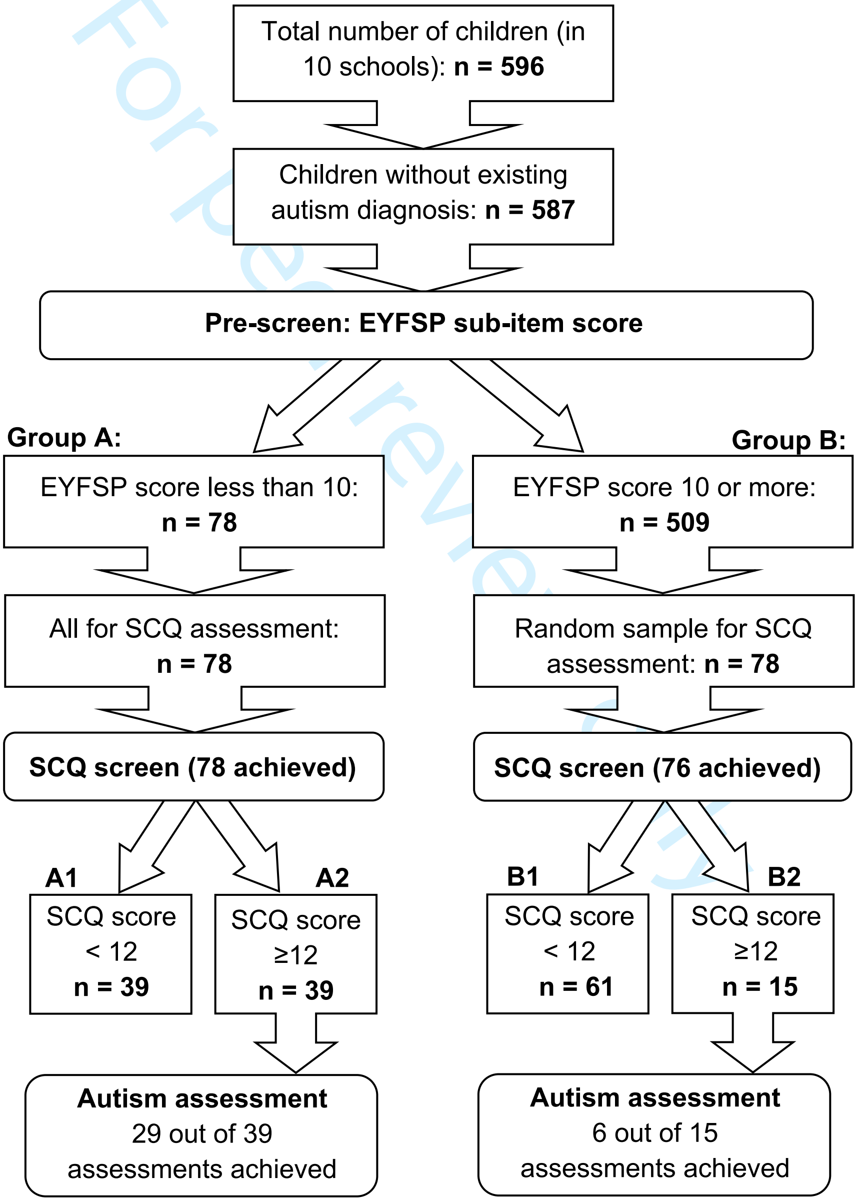

For peer review only - http://bmjopen.bmj.com/site/about/guidelines.xhtml

V6 18. 06. 2020

In order to check for false negatives we added an additional screening check where those children in the above groups given the SCQ scored below the threshold of 12 where their teacher filled in a narrative behaviour questionnaire mapping to the WHO research diagnostic criteria for ASD. (28) This yields a score of 0-12 to identify areas of concern in any of the 12 symptom groups for ASD (28). Any child who had already had a diagnosis on the Autism Spectrum from the local diagnostic services was also noted.

Finally, sensitivity analysis was carried out using a cut of 15 or the SCQ instead of 12 as this has been used in some studies (27)

The Autism Assessment

The assessments took place in those 10 schools in Bradford between September 2018 and July 2019. The assessment involved a team of three multidisciplinary staff drawn from a bank of child and adolescent mental health service (CAMHS) clinicians and educational psychologists. The assessment was completed in school in one day. One experienced clinician who was trained in the ADIR (28) carried out this parent based semi-structured interview with a parent or primary care giver. Two other professionals (usually an educational psychologist and a clinical psychologist or child psychiatrist) trained in the ADOS-2 (29) carried out this play/interaction based assessment with the child, using the most appropriate module depending on their developmental ability and language development. This was carried out by one person and observed by a second person and information shared during coding. One of the clinicians also observed the child in class with a bespoke ASD checklist. The clinicians went through a teacher based questionnaire related to the teacher's experiences of the child's skills and behaviour including the main symptoms of ASD using the World Health Organisation International Classification of Diseases Version 10 Research Diagnostic Criteria (30). Finally there was a consensus meeting with the three external assessors and the teacher identifying an overall consensus for the presence or absence of definite, possible or no difficulties in the 12 main research diagnostic criteria areas for Autism Spectrum Disorder diagnosis (28). In the afternoon each of the clinicians contributed to one single report using a range of sub-headings and organising material according to those sub-headings. This included a final consensus formulation, a description of strengths and difficulties and a range of recommendations. As agreed in ethical

For peer review only - http://bmjopen.bmj.com/site/about/guidelines.xhtml

V6 18. 06. 2020

approvals the report fell short of making an NHS diagnosis (since this was a research project). Where appropriate it was suggested that referral was made through appropriate local assessment pathways with the report. A range of other recommendations were made including referral elsewhere such as speech and language therapy assessment, physical health checks or a proposed assessment for an Education Health Care Plan, educational psychology assessment or a range. Given the breadth of experience of the assessing professionals and the teacher, a number of possible recommendations for assessment were possible.

Feasibility Outcomes

Feasibility outcomes were collected such as numbers consenting, attrition rates after consent, acceptability of assessment elements, recording of any language or interpreting issues and the acceptability and completion of questionnaires.

We carried qualitative interviews to obtain in-depth information from parents, teachers and clinicians about the acceptability, usefulness and real world provision of the assessment process.

Results

There were 596 children in the 10 schools, 587 were included in the study as 9 children from this cohort had a pre-existing autism diagnosis (Figure 1). 14 families decided that they did not want to be part of the study and did not consent. Two families moved to a different school.

For peer review only

For peer review only - http://bmjopen.bmj.com/site/about/guidelines.xhtml

V6 18. 06. 2020

510 children scored 10 or above on the Early Years Foundation Stage Profile and 86 children scored 9 or below (at risk children). Of the 86 children scoring 9 or below, 8 (9%) of these children already had a diagnosis on the autism spectrum and the remainder were given the Social Communication Questionnaire (SCQ) with threshold results for 12 and 15 reported below. (31).

Table 1 – Percentage of children who meet the threshold for ASD with threshold results for 12 and 15 in the SCQ

SCQ Scores (those score 12 or above)		
Autism Spectrum Disorder	Low EYFSP	Not Low EYFSP
Yes	9	0
No	20	6
Total	29	6
31% of those with Low EYSFP had diagnosis of ASD		

SCQ Scores (those score 15 or above)		
Autism Spectrum Disorder	Low EYFSP	Not Low EYFSP
Yes	8	0
No	13	3
Total	21	3
38% of those with Low EYSFP had diagnosis of ASD		

All but one of the children who were met the criteria for a diagnosis of ASD had a SCQ of 15 or above.

Of the 510 children screened 10 or above (i.e. a low risk score) on the Early Years Foundation Stage Profile 1 child had a diagnosis on the Autism Spectrum already. We randomised 15% of these children to carry out the SCQ and so 78 families completed this with 15 of them scoring 12 or above on the SCQ with 61 scoring under 12 and 2 lost follow ups. The comprehensive Autism assessments described were offered to 54 children scoring greater than or equal to 12 on the SCQ from the children scoring 9 or below on the EYFSP

For peer review only - http://bmjopen.bmj.com/site/about/guidelines.xhtml

with 39 carried out and with the random sub-group of those scoring 10 or above (n=15). Teachers to complete a comprehensive questionnaire based on the WHO research diagnostic criteria for ASD for 20 out of 39 children who scored 9 and below in EYFSP and less than 12 in SCQ as well as 33 out of 61 children who scored 10 or more in EYFSP and less than 12 in SCQ. We received a total of 53 questionnaires and none of them scored more than 2out of 12 on the research diagnostic criteria risk checklist, all below the level where a diagnosis of ASD would be likely. The large majority (88.68%) had 0 symptoms.

For peer review only

For peer review only - http://bmjopen.bmj.com/site/about/guidelines.xhtml

V6 18. 06. 2020

Those in group A (who score low on the EYFSP sub-score pre-screen) are more likely to be identified as potentially at risk of having ASD on the SCQ screening test compared to those in group B (those who do not score low on the EYFSP sub-score pre-screen); 50% of those in group A scored 12 or above on the SCQ, compared to 19% in group B (see table 2).

Table 2: Comparison between groups with low and high scores in EYFSP

	SCQ Screen		
EYFSP sub-score pre-screen	High SCQ	Low SCQ	Total
Group A	50%	50%	78
Group B	19%	81%	78
Total	35%	65%	156

Pearson chi2(1) = 16.3137 $p < 0.001$

Group A are those scoring low on the EYFSP sub-score pre-screen score

Group B are those not scoring low on the EYFSP sub-score pre-screen score

High SCQ are those that score at least 12 on SCQ (potential autism)

Low SCQ are those that score less than 12 on SCQ (not potential autism)

Families of children who scored 12 or more on the SCQ screening tool who were then offered a full autism assessment, are described in table 2. Those who score low on the EYFSP sub-score pre-screen and then who go onto score high on the SCQ score (indicating potential autism) are much more likely to be diagnosed with ASD after the full assessment, compared to those in group B (those who did not score low on the EYFSP sub-score pre-screen and then who go onto score high on the SCQ score).

For peer review only - http://bmjopen.bmj.com/site/about/guidelines.xhtml

V6 18. 06. 2020

- 31% of those in group A with a SCQ of 12 or more met the research diagnostic criteria for ASD diagnosis.
- None of those in group B with a SCQ of 12 or more met the research diagnostic criteria for ASD diagnosis.

Table 3 and 4 indicate the suggested referrals to other services that arose from the assessment, suggesting that this process may be useful in identifying children with a range of developmental problems and not simply those with ASD.

For peer review only

Table 3 Outcomes of assessments for those children with a SCQ score of 12 or above:

	**Group A2:**	**Group B2**	**Groups A2 & B2**
Referral to service	Pre-screen: Low EYFSP sub-score (n = 29)	Pre-screen: Not low EYFSP sub-score (n=6)	Total with autism assessment (n = 35)
Autism Spectrum Disorder	9 (31.0%)	0 (0%)	9 (25.7%)
Assessed Need for External (outside school system) support	22 (75.9%)	3 (50.0%)	25 (71.4%)
Assessed Need for Internal (within school system) support	29 (100%)	5 (83.3%)	34 (97.1%)
Assessed need for Internal or External Support	29 (100%)	6 (100%)	35 (100%)

For peer review only - http://bmjopen.bmj.com/site/about/guidelines.xhtml

Table 4: Recommendations from assessing clinicians about additional support needed for 35 assessed children

Enacted Onward Referral to service	**Group A:** Pre-screen: Low EYFSP sub-score (n = 29)	**Group B** Pre-screen: Not low EYFSP sub-score (n=6)	**Group A & B** Total with autism assessment (n = 35)
Autism Spectrum Disorder	9 (31.0%)	0 (0%)	9 (25.7%)
Speech and Language Therapy Assessment	16 (55.2%)	3 (50.0%)	19 (54.3%)
Nurture Group/Encouragement of social interaction/monitoring	12 (41.4%)	4 (66.7%)	16 (45.7%)
Learning Needs Assessment	4 (13.8%)	2 (33.3%)	6 (17.1%)
In school Lego Based Therapy	3 (10.3%)	0 (0%)	3 (8.6%)
Parent Support	3 (10.3%)	0 (0%)	3 (8.6%)
Dyslexia Assessment	3 (10.3%)	0 (0%)	3 (8.6%)
Dyscalculia Assessment/Maths Skills Support	1 (3.4%)	0 (0%)	1 (2.9%)
Ed Psych/Cognitive Assessment	9 (31.0%)	0 (0%)	9 (25.7%)
Formal EHCP triggered	5 (17.2%)	0 (0%)	5 (14.3%)
Visual Aids and/or vision assessment	5 (17.2%)	0 (0%)	5 (14.3%)
In school Creative Activities groups	3 (10.3%)	0 (0%)	3 (8.6%)
Gross Motor Skills Support	3 (10.3%)	1 (16.7%)	4 (11.4%)
Physical Health Check	2 (6.9%)	0 (0%)	2 (5.7%)
In school Social Story intervention	2 (6.9%)	0 (0%)	2 (5.7%)
New Adaptations in Classrooms	6 (20.7%)	0 (0%)	6 (17.1%)
Occupational Therapy assessment	1 (3.4%)	0 (0%)	1 (2.9%)
Other group support	1 (3.4%)	0 (0%)	1 (2.9%)
Attention Concentration Support	6 (20.7%)	1 (16.7%)	7 (20.0%)

For peer review only

For peer review only - http://bmjopen.bmj.com/site/about/guidelines.xhtml

V6 18. 06. 2020

We checked the GP records of those 35 children identified as having low (29 children) and not low (6 children) EYFSP scores and 12 or above on the SCQ. Only 4 of these children had previously had any READ codes recorded for intellectual disability, language delay or disorder, ADHD or ASD, all four being recorded as having speech delay or disorder of speech and language. Two of these four children were assessed in our study as meeting the criteria for ASD. The remaining 31 children with low and not low EYFSP and SCQ > 12 had no GP recorded Read codes but all 31 had additional needs newly identified in our assessments (see table 4). This shows that of the 35 children 31 would gain new interventions as a result of our assessment processes that they were not currently accessing. All 9 of the children who were newly diagnosed with ASD by this research were from an ethnic minority background.

Feasibility outcomes

All 10 schools approached participated in the study. From these 51 children identified as requiring an assessment and 32 children were finally assessed. 19 children were not assessed; 16 withdrew early on in the study and 2 left the school. We received back all initially requested from schools, both EYFSP and SCQs. Of the teacher questionnaires for children that were not identified as requiring an assessment 53 questionnaires of 55 were completed.

Qualitative findings

Associated qualitative research will be published separately. Feedback was requested clinicians, school staff, assessed children's parents and parents of children with a neurodevelopmental disorder from a patients' panel.

Both parents and clinicians were positive about school based assessment occurring largely in one day. This included the benefits of the child being in their normal routine and experiencing less anxiety than clinic visits. Parents were positive about not having to chase appointments and teachers positive about involvement in all assessments.

Clinicians valued multidisciplinary working and the positives of access to rich school based data. A SENCO from one of the school mentioned that *'I liked that everybody can come together because you are in one place, everybody that knows the child is there and then it is*

For peer review only - http://bmjopen.bmj.com/site/about/guidelines.xhtml

kind of written as a team around the child…'. Parents commented that including school in the assessment process had helped teaching staff to adapt teaching and support for the child promptly. Challenges identified included difficulties coordinating different professionals, children and parents together and last minute cancellations '*this process was highly dependent on administration both from the project and from school…*'. Other themes highlighted related to the diagnosis and a range of responses relating to concern that their child's problems may be minimised or that they might be stigmatised.

Discussion

This study has shown that it is feasible to carry out a larger study of a new assessment care pathway for neurodevelopmental problems across a district. The acceptability to families is relatively good, although some families had some concerns about the consequences of diagnosis or not. This suggests that care needs to be taken when considering the emotional consequences for the family. It is good practice to provide parenting support to families of children newly diagnosed with ASD and this should be a key part of new assessment pathways or future research.

In our trial the EYFSP pre-screen identified 13% of the pupil population (78 pupils scoring less than 10 on the EYFSP out of 587 pupils). Of this 13% of pupils half then go on to score high on the SCQ; so that approximately 6.5% of the pupil population would receive an autism assessment with the addition of the EYFSP pre-screen. This compares with 14% (11) in similar early life screening studies without a pre-screen stage. This has potential cost-effectiveness benefits that we were unable to test but should be key parts of future research.

A recent paper (32) suggests that, based on the cut off at 12, the sensitivity of the SCQ is 42% and the specificity 89%. Whilst we cannot accurately assess sensitivity in our study as we have not assessed all the children in the sample, we used teacher based questionnaires (with ASD research diagnostic criteria) in 33 children with normal EYFSP scores and low SCQ scores and none had more than 2 flagged areas of concern on the research diagnostic

For peer review only - http://bmjopen.bmj.com/site/about/guidelines.xhtml

criteria symptom list for ASD (5-6 is the threshold for diagnosis). This suggests that further research may reveal an improved sensitivity when EYFSP is used as a pre-screen before SCQ.

This study has shown that there may be promising alternatives to existing assessment pathways for ASD (i.e. the use of EYFSP sub-score as a pre-screen tool, prior to SCQ screening). Advantages to the clinical process include the fact that information can be gathered from the school with those who know the child best (parents/carers and teacher) in one day in an environment known to the child, which may give a more accurate assessment. Previous studies using screening instruments with similar sample sizes have found a third of the sample are lost to follow up (11). Our study has vastly lower attrition because of the close link with the clinical teams into schools where parents are in regular contact. The early identification of ASD means that children can access the best educational placement early and allows the local authority to plan its services and resources. It may resolve inequalities seen in previous studies where sections of the population do not come forward for assessment (17, 18).

This study identified a number of new children (n=9) with a diagnosis of ASD. This has enabled support to be established early. All of these children were from ethnic minorities suggesting that this process may be addressing inequalities in early diagnosis found in previous studies (17), although this would need further larger research to confirm. In other studies using the Social Communication Questionnaire, when children score above the threshold but do not have ASD, approximately 90% have a neurodevelopmental disorder or developmental problem of some sort requiring identification and support (33). In our study using the EYFSP this was 100% since all children had identified support needs.

The study was limited by its size suggesting further larger district level research with cost-effectiveness analysis needs to take place.

Acknowledgements

Thanks to all the professionals who helped with the assessments including, Dr Claudia Salt, Dr Emily Williams, Natalie Langley, Halimah Hafiz, Nabihah Kauser, Ronnie Hartley, Prakash Thapa, Dr Alice Lambert. Thanks also to a range of people for their help to make this

For peer review only - http://bmjopen.bmj.com/site/about/guidelines.xhtml

For peer review only

research possible including Professor John Wright, Dr David Sims, Rachael Vann, Sarah Oates, Amy Hart, Sophie Tully, Sarah Gates, Shelley Russell, Catarina Teige, Dani Varley, Dr Sue Lee and Rebecca Joy, Misbah Khan, Lydia Phillip.

A special thanks to Dr Stefan Williams and Dr Sujo Anathhanam who carried out earlier preparatory work prior to this study.

Contributorship statement:

Professor Barry Wright: Conceived of the presented idea, contributed to the design and delivery of the project and the writing up of the manuscript

Dr Konstantopoulou Kalliopi: Contributed to the design, delivery, data collection and the writing up of the manuscript

Kuldeep Sohal: Contributed to the design of the project and agreed with the manuscript's results and conclusions

Dr Brian Kelly: Contributed to the design of the project, completed the statistical analysis of the project and contributed to the writing of the manuscript

Dr Geoff Morgan: Contributed to the design, delivery of the project and agreed with the manuscript's results and conclusions

Cathy Hulin: Contributed to the design, overall organisation, data collection and writing up of the manuscript

Dr Sara Mansoor: Contributed to the design and delivery of the project

Professor Mark Mon-Williams: Contributed to the design of the project, agreed with the manuscript's results and conclusions

Competing interests:

There are no competing interests for any author

Funding statement

The work was conducted within infrastructure provided by the Centre for Applied Education Research (www.caer.org.uk), and funded by the Department for Education through the Bradford Opportunity Area. The views expressed are those of the author(s), and not necessarily those of the NHS, the Bradford Local Authority or the Department for Education.

Data sharing statement:

Data are available upon reasonable request.

For peer review only - http://bmjopen.bmj.com/site/about/guidelines.xhtml

V6 18. 06. 2020

For peer review only

For peer review only - http://bmjopen.bmj.com/site/about/guidelines.xhtml

V6 18. 06. 2020

References

1. Baird G, Simonoff E, Pickles A, Chandler S, Loucas T, Meldrum D, et al. Prevalence of disorders of the autism spectrum in a population cohort of children in South Thames: the Special Needs and Autism Project (SNAP). The lancet. 2006;368(9531):210-5.
2. Szatmari P, Georgiades S, Bryson S, Zwaigenbaum L, Roberts W, Mahoney W, et al. Investigating the structure of the restricted, repetitive behaviours and interests domain of autism. Journal of Child Psychology and Psychiatry. 2006;47(6):582-90.
3. Georgiades S, Szatmari P, Zwaigenbaum L, Duku E, Bryson S, Roberts W, et al. Structure of the autism symptom phenotype: A proposed multidimensional model. Journal of the American Academy of Child & Adolescent Psychiatry. 2007;46(2):188-96.
4. Saggers B, Klug D, Harper-Hill K, Ashburner J, Costley D, Clark T, et al. Australian autism educational needs analysis–What are the needs of schools, parents and students on the autism spectrum2018.
5. French L, Kennedy EM. Annual Research Review: Early intervention for infants and young children with, or at-risk of, autism spectrum disorder: a systematic review. Journal of Child Psychology and psychiatry. 2018;59(4):444-56.
6. Postorino V, Sharp WG, McCracken CE, Bearss K, Burrell TL, Evans AN, et al. A systematic review and meta-analysis of parent training for disruptive behavior in children with autism spectrum disorder. Clinical Child and Family Psychology Review. 2017;20(4):391-402.
7. Rogge N, Janssen J. The Economic Costs of Autism Spectrum Disorder: A Literature Review. Journal of autism and developmental disorders. 2019:1-28.
8. Saccà A, Cavallini F, Cavallini MC. Parents of Children with Autism Spectrum Disorder: a systematic review. Journal of Clinical & Developmental Psychology. 2019;1(3).
9. Saunders B, Sim J, Kingstone T, Baker S, Waterfield J, Bartlam B, et al. Saturation in qualitative research: exploring its conceptualization and operationalization. Quality & quantity. 2018;52(4):1893-907.
10. Zwaigenbaum L, Bauman ML, Choueiri R, Fein D, Kasari C, Pierce K, et al. Early identification and interventions for autism spectrum disorder: executive summary. Pediatrics. 2015;136(Supplement 1):S1-S9.
11. Janvier YM, Harris JF, Coffield CN, Louis B, Xie M, Cidav Z, et al. Screening for autism spectrum disorder in underserved communities: Early childcare providers as reporters. Autism. 2016;20(3):364-73.
12. Towle PO, Patrick PA. Autism spectrum disorder screening instruments for very young children: a systematic review. Autism research and treatment. 2016;2016.
13. Crane L, Chester JW, Goddard L, Henry LA, Hill E. Experiences of autism diagnosis: A survey of over 1000 parents in the United Kingdom. Autism. 2016;20(2):153-62.
14. Commission CQ. Care Quality Commission: The State of Health Care and Adult Social Care in England-HC 838: The Stationery Office; 2013.
15. Agency UGST. Early Years Foundation Stage Profile 2018 [18th December 2019]. Available from: https://assets.publishing.service.gov.uk/government/uploads/system/uploads/attachment_data/file/790580/EYFSP_Handbook_2019.pdf.
16. Wright B, Mon-Williams M, Kelly B, Williams S, Sims D, Mushtaq F, et al. Investigating the association between early years foundation stage profile scores and subsequent diagnosis of an autism spectrum disorder: a retrospective study of linked healthcare and education data. BMJ Paediatrics Open. 2019;3(1).
17. Kelly B, Williams S, Collins S, Mushtaq F, Mon-Williams M, Wright B, et al. The association between socioeconomic status and autism diagnosis in the United Kingdom for children aged 5–8 years of age: Findings from the Born in Bradford cohort. Autism. 2019;23(1):131-40.
18. Nowell KP, Brewton CM, Allain E, Mire SS. The influence of demographic factors on the identification of autism spectrum disorder: A review and call for research. Review Journal of Autism and Developmental Disorders. 2015;2(3):300-9.

For peer review only - http://bmjopen.bmj.com/site/about/guidelines.xhtml

V6 18. 06. 2020

19. Harris B, McClain MB, Haverkamp CR, Cruz RA, Benallie KJ, Benney CM. School-based assessment of autism spectrum disorder among culturally and linguistically diverse children. Professional Psychology: Research and Practice. 2019;50(5):323.
20. Chestnut SR, Wei T, Barnard-Brak L, Richman DM. A meta-analysis of the social communication questionnaire: Screening for autism spectrum disorder. Autism. 2017;21(8):920-8.
21. Dickerson J, Bird PK, McEachan RR, Pickett KE, Waiblinger D, Uphoff E, et al. Born in Bradford's Better Start: an experimental birth cohort study to evaluate the impact of early life interventions. BMC Public Health. 2016;16(1):711.
22. Shire K, Andrews E, Barber S, Bruce A, Corkett J, Hill L, et al. Starting School: a large-scale start of school assessment within the ?Born in Bradford? longitudinal cohort [version 1; peer review: 1 approved, 1 approved with reservations]. Wellcome Open Research. 2020;5(47).
23. Wiggins LD, Bakeman R, Adamson LB, Robins DL. The utility of the Social Communication Questionnaire in screening for autism in children referred for early intervention. Focus on Autism and Other Developmental Disabilities. 2007;22(1):33-8.
24. Lee L-C, David AB, Rusyniak J, Landa R, Newschaffer CJ. Performance of the Social Communication Questionnaire in children receiving preschool special education services. Research in Autism Spectrum Disorders. 2007;1(2):126-38.
25. Schanding GT, Nowell KP, Goin-Kochel RP. Utility of the social communication questionnaire-current and social responsiveness scale as teacher-report screening tools for autism spectrum disorders. Journal of Autism and Developmental Disorders. 2012;42(8):1705-16.
26. Corsello C, Hus V, Pickles A, Risi S, Cook Jr EH, Leventhal BL, et al. Between a ROC and a hard place: decision making and making decisions about using the SCQ. Journal of Child Psychology and Psychiatry. 2007;48(9):932-40.
27. Barnard-Brak L, Brewer A, Chesnut S, Richman D, Schaeffer AM. The sensitivity and specificity of the social communication questionnaire for autism spectrum with respect to age. Autism research : official journal of the International Society for Autism Research. 2016 Aug;9(8):838-45. PubMed PMID: 26608837. Epub 2015/11/27. eng.
28. Rutter M, Le Couteur A, Lord C. Autism diagnostic interview-revised. Los Angeles, CA: Western Psychological Services. 2003;29:30.
29. Lord C, Rutter, M., DiLavore, P., Risi, S., Gotham, K., & Bishop, S. Autism diagnostic observation schedule-2nd edition (ADOS-2),. Western Psycological Corporation 2012. Los Angeles, CA:.
30. Organization WH. The ICD-10 classification of mental and behavioural disorders: Diagnostic criteria for research: World Health Organization; 1993.
31. Oosterling I, Rommelse N, de Jonge M, van der Gaag RJ, Swinkels S, Roos S, et al. How useful is the Social Communication Questionnaire in toddlers at risk of autism spectrum disorder? Journal of child psychology and psychiatry, and allied disciplines. 2010 Nov;51(11):1260-8. PubMed PMID: 20626528. Epub 2010/07/16. eng.
32. Surén P, Saasen-Havdahl A, Bresnahan M, Hirtz D, Hornig M, Lord C, et al. Sensitivity and specificity of early screening for autism. BJPsych open. 2019 May 17;5(3):e41. PubMed PMID: 31530312. Pubmed Central PMCID: PMC6537444. Epub 2019/09/19. eng.
33. Chandler S, Charman T, Baird G, Simonoff E, Loucas T, Meldrum D, et al. Validation of the social communication questionnaire in a population cohort of children with autism spectrum disorders. Journal of the American Academy of Child & Adolescent Psychiatry. 2007;46(10):1324-32.

For peer review only - http://bmjopen.bmj.com/site/about/guidelines.xhtml

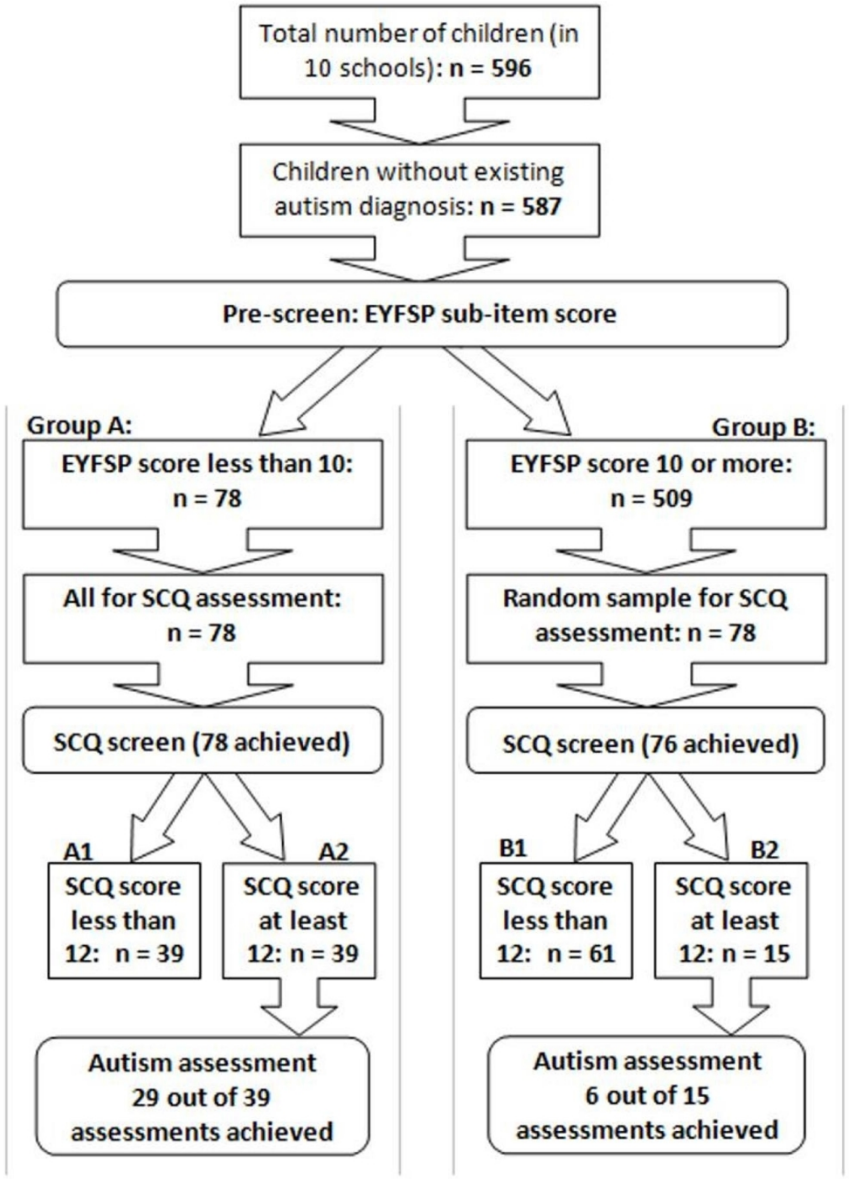

Study design

117x155mm (300 x 300 DPI)

Table 1 – Percentage of children who meet the threshold for ASD with threshold results for 12 and 15 in the SCQ

SCQ Scores (those score 12 or above)

Autism Spectrum Disorder	Low EYFSP	Not Low EYFSP
Yes	9	0
No	20	6
Total	29	6

31% of those with Low EYSFP had diagnosis of ASD

SCQ Scores (those score 15 or above)

Autism Spectrum Disorder	Low EYFSP	Not Low EYFSP
Yes	8	0
No	13	3
Total	21	3

38% of those with Low EYSFP had diagnosis of ASD

Table 2: Comparison between groups with low and high scores in EYFSP

	SCQ Screen		
EYFSP sub-score pre-screen	High SCQ	Low SCQ	Total
Group A	50%	50%	78
Group B	19%	81%	78
Total	35%	65%	156

Pearson chi2(1) = 16.3137 $p < 0.001$

Group A are those scoring low on the EYFSP sub-score pre-screen score

Group B are those not scoring low on the EYFSP sub-score pre-screen score

High SCQ are those that score at least 12 on SCQ (potential autism)

Low SCQ are those that score less than 12 on SCQ (not potential autism)

Table 3 Outcomes of assessments for those children with a SCQ score of 12 or above:

	Group A2:	Group B2	Groups A2 & B2

For peer review only

Referral to service	Pre-screen: Low EYFSP sub-score (n = 29)	Pre-screen: Not low EYFSP sub-score (n=6)	Total with autism assessment (n = 35)
Autism Spectrum Disorder	9 (31.0%)	0 (0%)	9 (25.7%)
Assessed Need for External (outside school system) support	22 (75.9%)	3 (50.0%)	25 (71.4%)
Assessed Need for Internal (within school system) support	29 (100%)	5 (83.3%)	34 (97.1%)
Assessed need for Internal or External Support	29 (100%)	6 (100%)	35 (100%)

For peer review only

Table 4: Recommendations from assessing clinicians about additional support needed for 35 assessed children

Enacted Onward Referral to service	**Group A:** Pre-screen: Low EYFSP sub-score (n = 29)	**Group B** Pre-screen: Not low EYFSP sub-score (n=6)	**Group A & B** Total with autism assessment (n = 35)
Autism Spectrum Disorder	9 (31.0%)	0 (0%)	9 (25.7%)
Speech and Language Therapy Assessment	16 (55.2%)	3 (50.0%)	19 (54.3%)
Nurture Group/Encouragement of social interaction/monitoring	12 (41.4%)	4 (66.7%)	16 (45.7%)
Learning Needs Assessment	4 (13.8%)	2 (33.3%)	6 (17.1%)
In school Lego Based Therapy	3 (10.3%)	0 (0%)	3 (8.6%)
Parent Support	3 (10.3%)	0 (0%)	3 (8.6%)
Dyslexia Assessment	3 (10.3%)	0 (0%)	3 (8.6%)
Dyscalculia Assessment/Maths Skills Support	1 (3.4%)	0 (0%)	1 (2.9%)
Ed Psych/Cognitive Assessment	9 (31.0%)	0 (0%)	9 (25.7%)
Formal EHCP triggered	5 (17.2%)	0 (0%)	5 (14.3%)
Visual Aids and/or vision assessment	5 (17.2%)	0 (0%)	5 (14.3%)
In school Creative Activities groups	3 (10.3%)	0 (0%)	3 (8.6%)
Gross Motor Skills Support	3 (10.3%)	1 (16.7%)	4 (11.4%)
Physical Health Check	2 (6.9%)	0 (0%)	2 (5.7%)
In school Social Story intervention	2 (6.9%)	0 (0%)	2 (5.7%)
New Adaptations in Classrooms	6 (20.7%)	0 (0%)	6 (17.1%)
Occupational Therapy assessment	1 (3.4%)	0 (0%)	1 (2.9%)
Other group support	1 (3.4%)	0 (0%)	1 (2.9%)
Attention Concentration Support	6 (20.7%)	1 (16.7%)	7 (20.0%)

For peer review only

STROBE Statement—Checklist of items that should be included in reports of ***cohort studies***

	Item No	Recommendation
**Title and abstract**	1	A systematic approach to school based assessments for Autism Spectrum Disorders to reduce inequalities? A feasibility study in ten primary schools.
		Objectives: This was a pilot study to explore whether the Early Years Foundation Stage Profile (EYFSP) carried out by teachers at the end of Reception year, followed by the Social Communication Questionnaire (SCQ) can lead to an earlier identification of children with Autism Spectrum Disorders (ASD), earlier access to intervention and reduce inequity in access to assessment and intervention.<br>Design: Pragmatic prospective cohort<br>Setting: 10 primary schools from the SHINE project in Bradford<br>Participants: 587 from 10 schools who transitioned from Reception to Year 1 in July 2017 and had the EYFSP completed were finally included in the study<br>Interventions: The assessment involved a team of three multidisciplinary staff who completed the Autism Diagnostic Interview Revised (ADI-R), the Autism Diagnostic Observation Schedule Version2 (ADOS-2), classroom observations with an ASD checklist, a teacher based ASD questionnaire and a final consensus meeting.<br>Primary outcome measure: NICE guideline compliant clinical diagnosis of ASD.<br>Secondary outcome measures: age of diagnosis, demographic data and feasibility parameters.<br>Results: Children who scored low on the EYFS were more likely to score above the SCQ threshold of 12(indicating potential autism), 50% compared to 19% of children not scoring low on the EYFS ($p < 0.001$). All children scoring above the SCQ received a full autism assessment; children who scored low on the EYFS were more likely to be diagnosed with autism (and other developmental issues) compared to those who did not score low on the EYFS.<br>Conclusions: We identified 9 new children with a diagnosis of ASD, all from ethnic minorities suggesting that this process may be addressing inequalities in early diagnosis found in previous studies. All children who scored above the threshold in the SCQ, required support and this was because the EYFSP questionnaire preceded it thereby including at risk children.
**Introduction**		
Background/rationale	2	Autism Spectrum Disorders (ASD) occur in approximately 1.6% of the UK population. Early identification and Early intervention has shown initial promise in improving outcomes. Whilst screening young children in early education settings has been attempted it identifies large numbers of children (14%) with relatively low numbers identified with ASD making cost effective whole population screening problematic. More nuanced approaches need to be developed. One promising approach would be to identify at risk populations and use screening and assessment processes within those groups. How to identify risk populations requires further research
Objectives	3	*Early Years Foundation Stage Profile (EYFSP)*<br>A large survey of parents in the UK describes late diagnosis in primary school despite symptoms being present from infancy and the Care Quality Commission found children with ASD having long waits for diagnosis and interventions. Recent studies suggest that using the Early Years Foundation Stage Profile may identify children with higher risk of having an ASD. The EYFSP is completed by teachers in England at the end of the reception year and scores 17 different domains of development in terms of whether a child is at an expected level, ahead or behind that level. It is used as a mechanism for flagging children who may need additional help in school and to benchmark UK school profiles.<br>*Equality of Access*<br>Recent work by the same group has also shown that the diagnosis of autism is less likely to be

For peer review only - http://bmjopen.bmj.com/site/about/guidelines.xhtml

made early in families from poor backgrounds or from families from ethnic minority groups showing inequalities reported elsewhere. This problem with equity of access would be well served by having a more widely available process for identifying children for neurodevelopmental disorder assessment as early as is practicable. One mechanism for improving equity of access is school based assessment.

Reasons for feasibility work

To plan a larger study it is necessary to gather feasibility information for improved assessment processes. We report a feasibility study of a two stage screening process involving the EYFSP followed by an established well validated ASD screening questionnaire, the Social Communication Questionnaire (SCQ). We sought to test the feasibility of a process where children went through this screening process and were then assessed more comprehensively for ASD in schools with education and health professionals working together over one day.

**Methods**		
Study design	4	This research was set within the larger Born in Bradford cohort research. We obtained consent from 10 primary schools in an existing project, the SHINE project.
Setting	5	The assessments took place in those 10 schools in Bradford between September 2018 and July 2019. The assessment involved a team of three multidisciplinary staff drawn from a bank of child and adolescent mental health service (CAMHS) clinicians and educational psychologists. The assessment was completed in school in one day. One experienced clinician who was trained in the ADIR carried out this parent based semi-structured interview with a parent or primary care giver. Two other professionals (usually an educational psychologist and a clinical psychologist or child psychiatrist) trained in the ADOS-2 carried out this play/interaction based assessment with the child, using the most appropriate module depending on their developmental ability and language development. This was carried out by one person and observed by a second person and information shared during coding. One of the clinicians also observed the child in class with a bespoke ASD checklist. The clinicians went through a teacher based questionnaire related to the teacher's experiences of the child's skills and behaviour including the main symptoms of ASD using the World Health Organisation International Classification of Diseases Version 10 Research Diagnostic Criteria. Finally there was a consensus meeting with the three external assessors and the teacher identifying an overall consensus for the presence or absence of definite, possible or no difficulties in the 12 main research diagnostic criteria areas for Autism Spectrum Disorder diagnosis. In the afternoon each of the clinicians contributed to one single report using a range of sub-headings and organising material according to those sub-headings. This included a final consensus formulation, a description of strengths and difficulties and a range of recommendations.
Participants	6	596 children in year 5 were available in 10 primary schools and we approached all of those who had received an Early Years Foundation Stage Profile scored by their teachers at the end of reception year in the summer of 2017.
Variables	7	As agreed in ethical approvals the report fell short of making an NHS diagnosis (since this was a research project). Where appropriate it was suggested that referral was made through appropriate local assessment pathways with the report. A range of other recommendations were made including referral elsewhere such as speech and language therapy assessment, physical health checks or a proposed assessment for an Education Health Care Plan, educational psychology assessment or a range. Given the breadth of experience of the assessing professionals and the teacher, a number of possible recommendations for assessment were possible.<br>*Feasibility Outcomes*

For peer review only

For peer review only - http://bmjopen.bmj.com/site/about/guidelines.xhtml

		Feasibility outcomes were collected such as numbers consenting, attrition rates after consent, acceptability of assessment elements, recording of any language or interpreting issues and the acceptability and completion of questionnaires.
Data sources/ measurement	8*	As described above
Bias	9	The assessments were performed by three independent clinicians using standardised tools according to the NICE guidelines criteria for an ASD assessment.
Study size	10	The teachers of children with low EYFSP scores and a 15% randomised sub-group of those with high scores (10 or more) completed a Social Communication Questionnaire (SCQ), which is a well-established validated autism screening questionnaire with good sensitivity and specificity scores. A threshold score of 12 or above on the SCQ was chosen, based on previous research; suggesting this is the best cut off for the optimum sensitivity to discriminate between children with and without ASD.
Quantitative variables	11	We carried qualitative interviews to obtain in-depth information from parents, teachers and clinicians about the acceptability, usefulness and real world provision of the assessment process.
Statistical methods	12	Data linkage allowed us to combine school and health data.<br>All those children and families with low EYFSP scores and above threshold SCQ (>12) were offered a NICE guideline compliant ASD Assessment, with additional clinical screening assessment for other developmental problems. A 15% randomised sub-group of those scoring high (10 or more) in EYFSP had the SCQ completed and those who scored high (10 or more) in EYFSP and 12 or above on the SCQ were then also assessed comprehensively in the same way. In order to check for false negatives we added an additional screening check where those children in the above groups given the SCQ scored below the threshold of 12 where their teacher filled in a narrative behaviour questionnaire mapping to the WHO research diagnostic criteria for ASD. This yields a score of 0-12 to identify areas of concern in any of the 12 symptom groups for ASD. Any child who had already had a diagnosis on the Autism Spectrum from the local diagnostic services was also noted.<br>Finally, sensitivity analysis was carried out using a cut of 15 or the SCQ instead of 12 as this has been used in some studies.
**Results**		
Participants	13*	There were 596 children in the 10 schools, 587 were included in the study as 9 children from this cohort had a pre-existing autism diagnosis. 14 families decided that they did not want to be part of the study and did not consent. Two families moved to a different school.
		Figures to be separately attached
Descriptive data	14*	There were 596 children in the 10 schools, 587 were included in the study as 9 children from this cohort had a pre-existing autism diagnosis (Figure 1). 14 families decided that they did not want to be part of the study and did not consent. Two families moved to a different school. 510 children scored 10 or above on the Early Years Foundation Stage Profile and 86 children scored 9 or below (at risk children). Of the 86 children scoring 9 or below, 8 (9%) of these children already had a diagnosis on the autism spectrum and the remainder were given the Social Communication Questionnaire (SCQ) with threshold results for 12 and 15 reported below.

For peer review only

SCQ Scores (those score 12 or above)		
Autism Spectrum Disorder	Low EYFSP	Not Low EYFSP
Yes	9	0
No	20	6
Total	29	6

31% of those with Low EYSFP had diagnosis of ASD

SCQ Scores (those score 15 or above)		
Autism Spectrum Disorder	Low EYFSP	Not Low EYFSP
Yes	8	0
No	13	3
Total	21	3

38% of those with Low EYSFP had diagnosis of ASD

All but one of the children who were met the criteria for a diagnosis of ASD had a SCQ of 15 or above.

Of the 510 children screened 10 or above (i.e. a low risk score) on the Early Years Foundation Stage Profile 1 child had a diagnosis on the Autism Spectrum already. We randomised 15% of these children to carry out the SCQ and so 78 families completed this with 15 of them scoring 12 or above on the SCQ with 61 scoring under 12 and 2 lost follow ups. The comprehensive Autism assessments described were offered to 54 children scoring greater than or equal to 12 on the SCQ from the children scoring 9 or below on the EYFSP with 39 carried out and with the random sub-group of those scoring 10 or above (n=15). Teachers to complete a comprehensive questionnaire based on the WHO research diagnostic criteria for ASD for 20 out of 39 children who scored 9 and below in EYFSP and less than 12 in SCQ as well as 33 out of 61 children who scored 10 or more in EYFSP and less than 12 in SCQ. We received a total of 53 questionnaires and none of them scored more than 2out of 12 on the research diagnostic criteria risk checklist, all below the level where a diagnosis of ASD would be likely. The large majority (88.68%) had 0 symptoms.

Outcome data	15*	See below
Main results	16	Those in group A (who score low on the EYFSP sub-score pre-screen) are more likely to be identified as potentially at risk of having ASD on the SCQ screening test compared to those in group B (those who do not score low on the EYFSP sub-score pre-screen); 50% of those in group A scored 12 or above on the SCQ, compared to 19% in group B (see table 1).

Table 1:

	SCQ Screen		
EYFSP sub-score pre-screen	High SCQ	Low SCQ	Total
Group A	50%	50%	78
Group B	19%	81%	78
Total	35%	65%	156

Pearson chi2(1) = 16.3137 p < 0.001

For peer review only - http://bmjopen.bmj.com/site/about/guidelines.xhtml

Group A are those scoring low on the EYFSP sub-score pre-screen score
Group B are those not scoring low on the EYFSP sub-score pre-screen score
High SCQ are those that score at least 12 on SCQ (potential autism)
Low SCQ are those that score less than 12 on SCQ (not potential autism)

Families of children who scored 12 or more on the SCQ screening tool who were then offered a full autism assessment, are described in table 2. Those who score low on the EYFSP sub-score pre-screen and then who go onto score high on the SCQ score (indicating potential autism) are much more likely to be diagnosed with ASD after the full assessment, compared to those in group B (those who did not score low on the EYFSP sub-score pre-screen and then who go onto score high on the SCQ score).

31% of those in group A with a SCQ of 12 or more met the research diagnostic criteria for ASD diagnosis.

None of those in group B with a SCQ of 12 or more met the research diagnostic criteria for ASD diagnosis.

Other analyses | 17

Table 3 and 4 indicate the suggested referrals to other services that arose from the assessment, suggesting that this process may be useful in identifying children with a range of developmental problems and not simply those with ASD.

Table 3 Outcomes of assessments for those children with a SCQ score of 12 or above:

Referral to service	Group A2: Pre-screen: Low EYFSP sub-score (n = 29)	Group B2 Pre-screen: Not low EYFSP sub-score (n=6)	Groups A2 & B2 Total with autism assessment (n = 35)
Autism Spectrum Disorder	9 (31.0%)	0 (0%)	9 (25.7%)
Assessed Need for External (outside school system) support	22 (75.9%)	3 (50.0%)	25 (71.4%)
Assessed Need for Internal (within school system) support	29 (100%)	5 (83.3%)	34 (97.1%)
Assessed need for Internal or External Support	29 (100%)	6 (100%)	35 (100%)

Table 4: Recommendations from assessing clinicians about additional support needed for 35 assessed children

	Group A:	Group B	Group A & B

For peer review only

Enacted Onward Referral to service	Pre-screen: Low EYFSP sub-score (n = 29)	Pre-screen: Not low EYFSP sub-score (n=6)	Total with autism assessment (n = 35)
Autism Spectrum Disorder	9 (31.0%)	0 (0%)	9 (25.7%)
Speech and Language Therapy Assessment	16 (55.2%)	3 (50.0%)	19 (54.3%)
Nurture Group/Encouragement of social interaction/monitoring	12 (41.4%)	4 (66.7%)	16 (45.7%)
Learning Needs Assessment	4 (13.8%)	2 (33.3%)	6 (17.1%)
In school Lego Based Therapy	3 (10.3%)	0 (0%)	3 (8.6%)
Parent Support	3 (10.3%)	0 (0%)	3 (8.6%)
Dyslexia Assessment	3 (10.3%)	0 (0%)	3 (8.6%)
Dyscalculia Assessment/Maths Skills Support	1 (3.4%)	0 (0%)	1 (2.9%)
Ed Psych/Cognitive Assessment	9 (31.0%)	0 (0%)	9 (25.7%)
Formal EHCP triggered	5 (17.2%)	0 (0%)	5 (14.3%)
Visual Aids and/or vision assessment	5 (17.2%)	0 (0%)	5 (14.3%)
In school Creative Activities groups	3 (10.3%)	0 (0%)	3 (8.6%)
Gross Motor Skills Support	3 (10.3%)	1 (16.7%)	4 (11.4%)
Physical Health Check	2 (6.9%)	0 (0%)	2 (5.7%)
In school Social Story intervention	2 (6.9%)	0 (0%)	2 (5.7%)
New Adaptations in Classrooms	6 (20.7%)	0 (0%)	6 (17.1%)
Occupational Therapy assessment	1 (3.4%)	0 (0%)	1 (2.9%)
Other group support	1 (3.4%)	0 (0%)	1 (2.9%)
Attention Concentration Support	6 (20.7%)	1 (16.7%)	7 (20.0%)

We checked the GP records of those 35 children identified as having low (29 children) and not low (6 children) EYFSP scores and 12 or above on the SCQ. Only 4 of these children had previously had any READ codes recorded for intellectual disability, language delay or disorder,

For peer review only

		ADHD or ASD, all four being recorded as having speech delay or disorder of speech and language. Two of these four children were assessed in our study as meeting the criteria for ASD. The remaining 31 children with low and not low EYFSP and SCQ > 12 had no GP recorded Read codes but all 31 had additional needs newly identified in our assessments (see table 4). This shows that of the 35 children 31 would gain new interventions as a result of our assessment processes that they were not currently accessing. All 9 of the children who were newly diagnosed with ASD by this research were from an ethnic minority background.
**Discussion**		
Key results	18	This study has shown that it is feasible to carry out a larger study of a new assessment care pathway for neurodevelopmental problems across a district.<br><br>In our trial the EYFSP pre-screen identified 13% of the pupil population (78 pupils scoring less than 10 on the EYFSP out of 587 pupils). Of this 13% of pupils half then go on to score high on the SCQ; so that approximately 6.5% of the pupil population would receive an autism assessment with the addition of the EYFSP pre-screen. This compares with 14% in similar early life screening studies without a pre-screen stage. This has potential cost-effectiveness benefits that we were unable to test but should be key parts of future research.<br>A recent paper suggests that, based on the cut off at 12, the sensitivity of the SCQ is 42% and the specificity 89%. Whilst we cannot accurately assess sensitivity in our study as we have not assessed all the children in the sample, we used teacher based questionnaires (with ASD research diagnostic criteria) in 33 children with normal EYFSP scores and low SCQ scores and none had more than 2 flagged areas of concern on the research diagnostic criteria symptom list for ASD (5-6 is the threshold for diagnosis). This suggests that further research may reveal an improved sensitivity when EYFSP is used as a pre-screen before SCQ.<br>This study has shown that there may be promising alternatives to existing assessment pathways for ASD (i.e. the use of EYFSP sub-score as a pre-screen tool, prior to SCQ screening). Advantages to the clinical process include the fact that information can be gathered from the school with those who know the child best (parents/carers and teacher) in one day in an environment known to the child, which may give a more accurate assessment. Previous studies using screening instruments with similar sample sizes have found a third of the sample are lost to follow up. Our study has vastly lower attrition because of the close link with the clinical teams into schools where parents are in regular contact. The early identification of ASD means that children can access the best educational placement early and allows the local authority to plan its services and resources. It may resolve inequalities seen in previous studies where sections of the population do not come forward for assessment.
Limitations	19	The study was limited by its size suggesting further larger district level research with cost-effectiveness analysis needs to take place.
Interpretation	20	This study identified a number of new children (n=9) with a diagnosis of ASD. This has enabled support to be established early. All of these children were from ethnic minorities suggesting that this process may be addressing inequalities in early diagnosis found in previous studies, although this would need further larger research to confirm. In other studies using the Social Communication Questionnaire, when children score above the threshold but do not have ASD, approximately 90% have a neurodevelopmental disorder or developmental problem of some sort requiring identification and support. In our study using the EYFSP this was 100% since all children had identified support needs.
Generalisability	21	The study gave promising results for a bigger study which could potentially include a larger

For peer review only - http://bmjopen.bmj.com/site/about/guidelines.xhtml

		number of participants
**Other information**		
Funding	22	The work was conducted within infrastructure provided by the Centre for Applied Education Research (www.caer.org.uk), and funded by the Department for Education through the Bradford Opportunity Area. The views expressed are those of the author(s), and not necessarily those of the NHS, the Bradford Local Authority or the Department for Education.

*Give information separately for exposed and unexposed groups.

Note: An Explanation and Elaboration article discusses each checklist item and gives methodological background and published examples of transparent reporting. The STROBE checklist is best used in conjunction with this article (freely available on the Web sites of PLoS Medicine at http://www.plosmedicine.org/, Annals of Internal Medicine at http://www.annals.org/, and Epidemiology at http://www.epidem.com/). Information on the STROBE Initiative is available at http://www.strobe-statement.org.

For peer review only

For peer review only - http://bmjopen.bmj.com/site/about/guidelines.xhtml

BMJ Open

A systematic approach to school based assessments for Autism Spectrum Disorders to reduce inequalities: A feasibility study in ten primary schools.

Journal:	*BMJ Open*
Manuscript ID	bmjopen-2020-041960.R1
Article Type:	Original research
Date Submitted by the Author:	03-Oct-2020
Complete List of Authors:	Wright, Barry; Hull York Medical School University of York<br>Konstantopoulou, Kalliopi; Leeds Community Healthcare NHS Trust, Child and Adolescent Psychiatry<br>Sohal, Kuldeep; Bradford Institute for Health Research<br>Kelly, Brian; Bradford Institute for Health Research<br>Morgan, Geoff; Educational Psychology Team, City of Bradford, Metropolitan District Council<br>Hulin, Cathy; Bradford Institute for Health Research<br>Mansoor, Sara; Bradford District Care NHS Foundation Trust<br>Mon-Williams, Mark; University of Leeds Department of Psychology
**Primary Subject Heading**:	Paediatrics
Secondary Subject Heading:	Mental health
Keywords:	Community child health < PAEDIATRICS, Child & adolescent psychiatry < PSYCHIATRY, Developmental neurology & neurodisability < PAEDIATRICS

SCHOLARONE™
Manuscripts

I, the Submitting Author has the right to grant and does grant on behalf of all authors of the Work (as defined in the below author licence), an exclusive licence and/or a non-exclusive licence for contributions from authors who are: i) UK Crown employees; ii) where BMJ has agreed a CC-BY licence shall apply, and/or iii) in accordance with the terms applicable for US Federal Government officers or employees acting as part of their official duties; on a worldwide, perpetual, irrevocable, royalty-free basis to BMJ Publishing Group Ltd ("BMJ") its licensees and where the relevant Journal is co-owned by BMJ to the co-owners of the Journal, to publish the Work in this journal and any other BMJ products and to exploit all rights, as set out in our licence.

The Submitting Author accepts and understands that any supply made under these terms is made by BMJ to the Submitting Author unless you are acting as an employee on behalf of your employer or a postgraduate student of an affiliated institution which is paying any applicable article publishing charge ("APC") for Open Access articles. Where the Submitting Author wishes to make the Work available on an Open Access basis (and intends to pay the relevant APC), the terms of reuse of such Open Access shall be governed by a Creative Commons licence – details of these licences and which Creative Commons licence will apply to this Work are set out in our licence referred to above.

Other than as permitted in any relevant BMJ Author's Self Archiving Policies, I confirm this Work has not been accepted for publication elsewhere, is not being considered for publication elsewhere and does not duplicate material already published. I confirm all authors consent to publication of this Work and authorise the granting of this licence.

For peer review only

V6 18. 06. 2020

A systematic approach to school based assessments for Autism Spectrum Disorders to reduce inequalities: A feasibility study in ten primary schools.

Professor Barry Wright[1]
Dr Konstantopoulou Kalliopi[2]
Kuldeep Sohal [3]
Dr Brian Kelly[3]
Dr Geoff Morgan [4]
Cathy Hulin[3]
Dr Sara Mansoor[5]
Professor Mark Mon-Williams[6]

1 Hull York Medical School, University of York

2 Leeds Community Healthcare NHS Foundation Trust

3 Bradford Institute for Health Research

4 Educational Psychology Team, City of Bradford, Metropolitan District Council

5 Bradford District Care NHS Foundation Trust

6 Department of Psychology, University of Leeds.

Corresponding author email address: artekon84@gmail.com

For peer review only

V6 18. 06. 2020

Abstract:

Objectives: This was a pilot study to explore whether the Early Years Foundation Stage Profile (EYFSP) carried out by UK teachers within 'Reception' year, combined with the Social Communication Questionnaire (SCQ) can lead to an earlier identification of children with Autism Spectrum Disorders (ASD), earlier access to intervention, and reduce inequity in access to assessment and intervention.

Design: Pragmatic prospective cohort.

Setting: Ten primary schools from the SHINE project in Bradford.

Participants: Five hundred eighty seven (587) pupils from ten schools who transitioned from Reception to Year 1 in July 2017 and had the EYFSP completed were included in the final study.

Interventions: The assessment involved: a multidisciplinary team of three staff who completed the Autism Diagnostic Interview Revised (ADI-R), the Autism Diagnostic Observation Schedule Version2 (ADOS-2), classroom observations with an ASD checklist, a teacher based ASD questionnaire, and a final consensus meeting.

Primary outcome measure: NICE guideline compliant clinical diagnosis of ASD.

Secondary outcome measures: age of diagnosis, demographic data and feasibility parameters.

Results: Children with low scores on the EYFS were more likely to score above the SCQ threshold of 12 indicating potential autism (50% compared to 19% of children with high scores on the EYFS ($p < 0.001$)). All children scoring above SCQ threshold received a full autism assessment; children who scored low on the EYFS were more likely to be diagnosed with autism (and other developmental issues) compared to those who did not.

Conclusions: We identified nine new children with a diagnosis of ASD, all from ethnic minorities suggesting that this process may be addressing the inequalities in early diagnosis found in previous studies. All children who scored above the SCQ threshold required support (i.e. had a neurodevelopmental disorder), indicating the EYFSP questionnaire captured 'at risk' children.

For peer review only - http://bmjopen.bmj.com/site/about/guidelines.xhtml

V6 18. 06. 2020

Strengths and limitations of the study:

- Consent was sought from all parents regardless of language by flexible use of interpreters.
- Education and Health data was shared yielding significant benefits
- We conducted the SCQ (threshold of 12) with children who scored ≤ 9 in the EYFSP and a random sub-sample from the high EYFSP group (15% of children ≥ 10)
- All children with a score of ≥ 12 on the SCQ received a detailed comprehensive ASD assessment and the rest had a teachers' screening questionnaire
- Any child who had already had a diagnosis on the Autism Spectrum from the local diagnostic services was also noted

For peer review only

For peer review only - http://bmjopen.bmj.com/site/about/guidelines.xhtml

V6 18. 06. 2020

Introduction

What is Autism

Autism Spectrum Disorders (ASD) occur in approximately 1.6% of the UK population (1). ASD is a neurodevelopmental condition that often includes a range of repetitive behaviours, preoccupations and interests (2), and large developmental differences in social communication relative to neuro-typically developing individuals (3). ASD leads to a need for different approaches to education (4) and parenting (5), (6), which can be costly for local authorities (7) and stressful for parents and family (8); (9).

Early identification

Early identification and early intervention has shown promise in improving outcomes (10), (5). Screening young children in early education settings has been attempted, but captures relatively low numbers of children with ASD (11) despite large numbers (14%) being identified at risk. This has made cost effective whole population screening problematic (12), and there is a need for more nuanced approaches. The ability to use routine data to identify 'at risk' populations remains the holy grail of autism assessment (12). The need for such approaches was shown within a large survey of parents in the UK who reported receiving a diagnosis late in primary school despite symptoms being present from infancy (13). This was confirmed by the Care Quality Commission who reported that children with ASD experience long waits for diagnosis and interventions (14).

Early Years Foundation Stage Profile (EYFSP)

Recent studies suggest that using the Early Years Foundation Stage Profile (15) may identify children with higher risk of having an ASD (16). The EYFSP is completed by teachers in England at the end of the reception year and scores 17 different domains of development in terms of whether a child is at an expected level, ahead, or behind that level. It is used as a mechanism for flagging children who may need additional help in school and to benchmark UK school profiles (15).

For peer review only

For peer review only - http://bmjopen.bmj.com/site/about/guidelines.xhtml

V6 18. 06. 2020

Equality of Access

Recent work has shown that the diagnosis of autism is less likely to be made early in families from poor backgrounds or from families from ethnic minority groups (17) - reflecting inequalities reported elsewhere (18). This problem with equity of access could be addressed by having a more widely available process for identifying children with neurodevelopmental disorder as early as possible. One mechanism for improving equity of access is school based assessments (19).

Reasons for feasibility work

In order to plan a larger study, it is necessary to gather feasibility information for improved assessment processes. We report a feasibility study of a two stage screening process involving the EYFSP followed by an established well validated ASD screening questionnaire = the Social Communication Questionnaire (20). We sought to test the feasibility of a process where children went through this screening process and were then assessed more comprehensively for ASD *in schools* with education and health professionals working together over the course of one day.

Methodology

Background

The research was set within the larger Born in Bradford cohort study (21). We obtained consent from 10 primary schools in an existing consortium, the SHINE partnership. The SHINE group is a group of ten primary schools that act as a testbed for new approaches to improve services, reduce inequalities, and test innovations (22). We obtained ethical approval from University of Leeds and Bradford Teaching Hospitals NHS Foundation Trust (IRAS Number: 233328).

Consent

All parents were approached with a family information leaflet and a consent form. A researcher was available by phone, email, or face-to-face for those wishing to discuss the project further. Interpreters were available because many of the population had a first language that was not English.

For peer review only - http://bmjopen.bmj.com/site/about/guidelines.xhtml

V6 18. 06. 2020

Design

Five hundred and ninety six (596) children in Year 5 were available in 10 primary schools and we approached all of those who had received an Early Years Foundation Stage Profile scored by their teachers at the end of reception year in the summer of 2017.

The study was designed to test feasibility for a larger study.

Measures

A screening measure to identify children at risk was derived from five items of the EYFSP carried out by teachers at the end of reception year. The measure was taken from the four main symptom areas defined in the research diagnostic criteria for ASD – namely, social reciprocity, language and communication, imagination delays, and repetitive and stereotyped patterns of behaviour. This is described in more detail in a previous study (16). EYFSP assessment scores are recorded for children in Reception who are aged from 4 to 5 years. The assessments conducted by the clinicians occurred in Year 1 when children are typically aged 5 to 6 years of age. We chose a score threshold of 9 which a previous study found to be significantly (statistically) associated with over 50 times the risk of autism: 52.7 (95% CI: 25.2 - 110.5). (16). Children were dichotomously grouped into 'low' (≤ 9) and 'high' (≥ 10) scorers.

The teachers of children with low EYFSP scores and a 15% randomised sub-group of those with high scores (≥ 10) completed a Social Communication Questionnaire (SCQ) (23), which is a well-established validated autism screening questionnaire with good sensitivity and specificity scores. In previous studies the SCQ has been found to be helpful in identifying young children with ASD (24). A threshold score of ≥ 12 on the SCQ was chosen based on previous research (25), with claims that this is the best threshold with the optimum sensitivity to discriminate between children with and without ASD (26). A sensitivity analysis was prospectively agreed for the threshold of >=15

Methods

Data linkage allowed us to combine school and health data (26).

For peer review only - http://bmjopen.bmj.com/site/about/guidelines.xhtml

All those children and families with low EYFSP scores and above threshold SCQ (>12) were offered a NICE guideline compliant ASD Assessment, with additional clinical screening assessment for other neurodevelopmental problems including speech and language difficulties, learning difficulties, physical health problems, anxiety, and low self-esteem. A 15% randomised sub-group of those scoring high (≥ 10) in EYFSP had the SCQ completed and those who scored ≥ 10 in EYFSP and ≥ 12 on the SCQ were then also assessed comprehensively in the same way. There were 596 children in the 10 schools, 587 were included in the study as nine children from this cohort had a pre-existing autism diagnosis. Fourteen (14) families decided that they did not want to be part of the study and did not consent. Two families moved to a different school (Figure 1).

Insert Figure 1 here

In order to check for false negatives, we added an additional screening check for the children in the above groups. In cases where the SCQ was scored below the threshold of 12, teachers filled in a narrative behaviour questionnaire mapping to the WHO research diagnostic criteria for ASD (28). This yields a score of 0-12 to identify areas of concern in any of the twelve symptom groups for ASD (28). Any child who had already had a diagnosis on the Autism Spectrum from the local diagnostic services was also noted.

Finally, sensitivity analysis was carried out using a cut off 15 of the SCQ instead of 12 as this has been used in some studies (27).

Patient and Public Involvement

There has been strong involvement and co-design of this research through the Born in Bradford governors' group, the Connected Yorkshire Patient and Public Involvement panel, SHINE schools, parents, young people, and other stakeholders. They have been supportive in the preparatory workshops, feasibility phases and information design of the study. We consulted with the Connected Yorkshire Patient and Public Involvement panel throughout the life cycle of this study who acknowledged the importance to improve the pathway to earlier diagnosis of Child ASD to improve children's health and wellbeing outcomes. The panel consists of parents that have children diagnosed with Child ASD or have children that are on the neurodevelopmental disorder care pathways. Some of the discussions focussed

For peer review only - http://bmjopen.bmj.com/site/about/guidelines.xhtml

V6 18. 06. 2020

on the stigma within certain communities in Bradford with certain mental health issues which result in parents not acknowledging the child's health issues and seeking diagnosis earlier or seeking the appropriate support across health or the education sectors.

We have also extensively engaged with the Headteachers at the Bradford SHINE primary schools and other school staff who helped to inform parents of the study and in the recruitment phase. The Bradford SHINE schools were actively involved in the design and implementation phase and wish to acknowledge our gratitude in the supporting, co-designing, and active involvement in this study.

We disseminated information on the study via the local radio stations including Bradford Ramadan, BBC Radio 4 and via a following website to inform individuals of the research that is being undertaken in the region.

Website: https://caer.org.uk/autism-spectrum-conditions/

We have also disseminated the results of the study via dedicated workshops at the Born in Bradford event in September 2019 and a further workshop in January 2020. These workshops consisted of a broad range of professional stakeholders from health and education across the region that are involved in the care pathway as well as public representation. The discussions have evolved to how the research study could be scaled across the region.

The Autism Assessment

The assessments took place in the 10 schools in Bradford between September 2018 and July 2019. The assessment involved a team of three multidisciplinary staff drawn from a bank of child and adolescent mental health service (CAMHS) clinicians, and educational psychologists. The assessment was completed in school in one day. One experienced clinician who was trained in the ADI-R (28) carried out the parent based semi-structured interview with a parent or primary care giver. Two other professionals (usually an educational psychologist and a clinical psychologist or child psychiatrist) trained in the

For peer review only - http://bmjopen.bmj.com/site/about/guidelines.xhtml

V6 18. 06. 2020

ADOS-2 (29) carried out the play/interaction based assessment with the child, using the most appropriate module depending on the child's developmental ability and language development. The assessment was carried out by one person and observed by a second with information shared during coding. One of the clinicians also observed the child in class with a bespoke ASD checklist. The clinicians went through a teacher based questionnaire related to the teacher's experiences of the child's skills and behaviour, including the main symptoms of ASD, using the World Health Organisation International Classification of Diseases Version 10 Research Diagnostic Criteria (30). Finally there was a consensus meeting with the three external assessors and the teacher, identifying an overall consensus for the presence or absence of definite, possible or no difficulties in the twelve main research diagnostic criteria areas for Autism Spectrum Disorder diagnosis (28). In the afternoon, each of the clinicians contributed to one single report using a range of sub-headings, and organised material according to those sub-headings. This included a final consensus formulation, a description of strengths and difficulties and a range of recommendations. As agreed in ethical approvals the report fell short of making an NHS diagnosis (since this was a research project). It was suggested where appropriate that referral was made through appropriate local assessment pathways with the report. A range of other recommendations were made including referral elsewhere (e.g. speech and language therapy assessment), physical health checks or a proposed assessment for an Education Health Care Plan, educational psychology assessment or a range of actions. Given the breadth of experience of the assessing professionals and the teacher, a number of possible recommendations for assessment were possible.

Feasibility Outcomes

Feasibility outcomes were collected. These included numbers consenting, attrition rates after consent, acceptability of assessment elements, recording of any language or interpreting issues and the acceptability and completion of questionnaires.

We conducted qualitative interviews to obtain in-depth information from parents, teachers and clinicians about the acceptability, usefulness and real-world provision of the assessment process.

For peer review only - http://bmjopen.bmj.com/site/about/guidelines.xhtml

V6 18. 06. 2020

Results

Five hundred and ten (510) children scored ≥ 10 on the Early Years Foundation Stage Profile and 86 children scored ≤ 9 (at risk children). Of the 86 children scoring ≤ 9, eight (9%) already had a diagnosis on the autism spectrum and the remainder were given the Social Communication Questionnaire (SCQ) with threshold results for ≥ 12 and 15 reported below (31).

Table 1 – Percentage of children who met the threshold for ASD with threshold results ≥ 12 and 15 in the SCQ

**SCQ Scores (those score 12 or above)**		
**Autism Spectrum Disorder**	***Low EYFSP***	***Not Low EYFSP***
Yes	9	0
No	20	6
**Total**	**29**	**6**
31% of those with Low EYSFP had diagnosis of ASD		
**SCQ Scores** (those score 15 or above)		
**Autism Spectrum Disorder**	***Low EYFSP***	***Not Low EYFSP***
Yes	8	0
No	13	3
**Total**	**21**	**3**
38% of those with Low EYSFP had diagnosis of ASD		

All but one of the children who were met the criteria for a diagnosis of ASD had a SCQ of 15 or above meaning that 11 assessments were needed to identify one extra child with ASD.

Of the 510 children with ≥ 10 on the Early Years Foundation Stage Profile (i.e. a low risk score), one child already had a diagnosis on the Autism Spectrum. We conducted the SCQ on a randomised sample (15%) of these children. Seventy eight families completed the SCQ with fifteen scoring ≥ 12 on the SCQ, 61 scoring ≤ 11, and two lost during follow up. The comprehensive Autism assessments described were offered to 54 children scoring ≥ 12 on the SCQ from the children scoring 9 or below on the EYFSP with 39 carried out and with the random sub-group of those scoring 10 or above (n=15). Teachers completed a

For peer review only - http://bmjopen.bmj.com/site/about/guidelines.xhtml

comprehensive questionnaire based on the WHO research diagnostic criteria for ASD for 20 out of 39 children who scored ≤ 9 in EYFSP and ≤ 11 in SCQ, as well as 33 out of 61 children who scored ≥ 10 in EYFSP and ≤ 11 in SCQ. We received a total of 53 questionnaires and none of them scored more than 2 out of 12 on the research diagnostic criteria risk checklist, all below the level where a diagnosis of ASD would be likely. The large majority (88.68%) had zero indicators.

Those in group A (who scored low on the EYFSP sub-score pre-screen) were more likely to be identified as potentially at risk of having ASD on the SCQ screening test compared to those in group B (those who did not score low on the EYFSP sub-score pre-screen); 50% of those in group A scored ≥ 12 on the SCQ, compared to 19% in group B (see table 2).

Table 2: Comparison between EYFSP and SCQ groups

	SCQ Screen		
EYFSP sub-score pre-screen	**High SCQ**	**Low SCQ**	Total
Group A	50%	50%	78
Group B	19%	81%	78
**Total**	**35%**	**65%**	**156**

Pearson chi2(1) = 16.3137 $p < 0.001$

Group A are those scoring low on the EYFSP sub-score pre-screen score

Group B are those not scoring low on the EYFSP sub-score pre-screen score

High SCQ are those that score at least 12 on SCQ (potential autism)

Low SCQ are those that score less than 12 on SCQ (not potential autism)

Families of children who scored ≥ 12 on the SCQ screening tool who were then offered a full autism assessment, are described in Table 2. Those who scored low on the EYFSP sub-score pre-screen and then scored high on the SCQ score (indicating potential autism spectrum

For peer review only - http://bmjopen.bmj.com/site/about/guidelines.xhtml

V6 18. 06. 2020

disorder) were much more likely to be diagnosed with ASD after the full assessment, compared to those in group B (those who did not score low on the EYFSP sub-score pre-screen and then scored high on the SCQ score). Thirty one percent of those in group A with a SCQ of ≥ 12 met the research diagnostic criteria for ASD diagnosis. None of those in group B with a SCQ of ≥ 12 met the research diagnostic criteria for ASD diagnosis.

Table 3 and 4 indicate the suggested referrals to other services that arose from the assessment, indicating that this process may be useful in identifying children with a range of neurodevelopmental problems and not simply those with ASD.

Table 3 Assessment outcomes according to risk groups for children scoring at least 12 on the SCQ (potential autism):

	Group A2	Group B2	Groups A2 & B2
Referral to service	*Pre-screen: Low EYFSP sub-score (n = 29)*	*Pre-screen: Not low EYFSP sub-score (n=6)*	*Total with autism assessment (n = 35)*
Autism Spectrum Disorder	9 (31.0%)	0 (0%)	9 (25.7%)
Assessed Need for External (outside school system) support	22 (75.9%)	3 (50.0%)	25 (71.4%)
Assessed Need for Internal (within school system) support	29 (100%)	5 (83.3%)	34 (97.1%)
Assessed need for Internal or External Support	29 (100%)	6 (100%)	35 (100%)

Group A2 are those scoring low on the EYFSP sub-score pre-screen score and scoring at least 12 on SCQ (potential autism)

Group B2 are those not scoring low on the EYFSP sub-score pre-screen score and scoring at least 12 on SCQ (potential autism)

For peer review only - http://bmjopen.bmj.com/site/about/guidelines.xhtml

V6 18. 06. 2020

Table 4: Recommendations from assessing clinicians about additional support needed for 35 assessed children

Enacted Onward Referral to service	Group A2<br>Pre-screen: Low EYFSP sub-score (n = 29)	Group B2<br>Pre-screen: Not low EYFSP sub-score (n=6)	Group A2 & B2<br>Total with autism assessment (n = 35)
**Autism Spectrum Disorder**	9 (31.0%)	0 (0%)	9 (25.7%)
**Speech and Language Therapy Assessment**	16 (55.2%)	3 (50.0%)	19 (54.3%)
**Nurture Group/Encouragement of social interaction/monitoring**	12 (41.4%)	4 (66.7%)	16 (45.7%)
**Learning Needs Assessment**	4 (13.8%)	2 (33.3%)	6 (17.1%)
**In school Lego Based Therapy**	3 (10.3%)	0 (0%)	3 (8.6%)
**Parent Support**	3 (10.3%)	0 (0%)	3 (8.6%)
**Dyslexia Assessment**	3 (10.3%)	0 (0%)	3 (8.6%)
**Dyscalculia Assessment/Maths Skills Support**	1 (3.4%)	0 (0%)	1 (2.9%)
**Ed Psych/Cognitive Assessment**	9 (31.0%)	0 (0%)	9 (25.7%)
**Formal EHCP triggered**	5 (17.2%)	0 (0%)	5 (14.3%)
**Visual Aids and/or vision assessment**	5 (17.2%)	0 (0%)	5 (14.3%)
**In school Creative Activities groups**	3 (10.3%)	0 (0%)	3 (8.6%)
**Gross Motor Skills Support**	3 (10.3%)	1 (16.7%)	4 (11.4%)
**Physical Health Check**	2 (6.9%)	0 (0%)	2 (5.7%)
**In school Social Story intervention**	2 (6.9%)	0 (0%)	2 (5.7%)
**New Adaptations in Classrooms**	6 (20.7%)	0 (0%)	6 (17.1%)
**Occupational Therapy assessment**	1 (3.4%)	0 (0%)	1 (2.9%)
**Other group support**	1 (3.4%)	0 (0%)	1 (2.9%)
**Attention Concentration Support**	6 (20.7%)	1 (16.7%)	7 (20.0%)

Group A2 are those scoring low on the EYFSP sub-score pre-screen score, and scoring at least 12 on SCQ (potential autism)

Group B2 are those not scoring low on the EYFSP sub-score pre-screen score, and scoring at least 12 on SCQ (potential autism)

V6 18. 06. 2020

We checked the GP records of those 35 children identified as having low (29 children) and not low (6 children) EYFSP scores and ≥ 12 on the SCQ. Only four of these children had previously had any READ codes recorded for intellectual disability, language delay or disorder, ADHD or ASD, all four being recorded as having speech delay or disorder of speech and language. Two of these four children were assessed in our study as meeting the criteria for ASD. The remaining 31 children with low and not low EYFSP and SCQ > 12 had no GP recorded Read codes but all 31 had additional needs that were newly identified in our assessments (see table 4). This shows that of the thirty five children, 31 would gain new interventions as a result of our assessment processes that they were not currently accessing. All nine of the children who were newly diagnosed with ASD by this research were from an ethnic minority background. There were six boys and three girls that were diagnosed with ASD. From the six boys, there were three of Pakistani origin, two of Bangladeshi origin and one gypsy/traveller origin. From the three girls that were diagnosed with ASD, two are of Pakistani origin and one is of Bangladeshi heritage.

Qualitative findings

Associated qualitative research will be published separately. Feedback was requested from clinicians, school staff, assessed children's parents, and parents of children with a neurodevelopmental disorder from a patients' panel.

Both parents and clinicians were positive about school based assessment occurring (largely) in one day. This included the benefits of the child being in their normal routine and experiencing less anxiety than clinic visits. Parents were positive about not having to chase appointments and teachers were positive about involvement in all assessments.

Clinicians valued multidisciplinary working and the positives of access to rich school based data. A SENCO from one of the school mentioned that *"I liked that everybody can come together because you are in one place, everybody that knows the child is there and then it is kind of written as a team around the child…".* Parents commented that including school in the assessment process had helped teaching staff to adapt teaching and support for the child promptly. Challenges identified included difficulties coordinating different

For peer review only - http://bmjopen.bmj.com/site/about/guidelines.xhtml

professionals, children and parents together and last minute cancellations "*this process was highly dependent on administration both from the project and from school…*". Other themes highlighted related to the diagnosis and a range of responses relating to concern from a parent that their child's problems may be minimised or that they might be stigmatised.

Discussion

This study has shown that it is feasible to carry out a larger study of a new assessment care pathway for neurodevelopmental problems across a district. We found that schools were very willing to take part in the study, and showed great interest in early identification of children with autism, and other support needs. All schools we approached in Bradford agreed to take part and facilitate the study. Teachers were supportive, completing 53 of 55 questionnaires about the children who did not receive the full autism assessment. The acceptability to families is relatively good, although some families withdrew from the study and some had concerns about the consequences of their child receiving a diagnosis of ASD. This suggests that care needs to be taken when considering the emotional consequences for the family. It is good practice to provide parenting support to families of children newly diagnosed with ASD and this should be a key part of new assessment pathways or future research.

In our trial, the EYFSP pre-screen identified 13% of the pupil population (78 pupils scoring less than 10 on the EYFSP out of 587 pupils). From this population, half scored highly on the SCQ such that approximately 6.5% of the population received an autism identification with the addition of the EYFSP pre-screen. This compares with 14% (11) in similar early life screening studies without a pre-screen stage. This has potential cost-effective benefits that we were unable to test but should be key parts of future research.

A recent paper (32) suggests an SCQ threshold of 12, with a sensitivity of 42% and specificity 89%. Other authors have used 15 (31). Our analysis shows 35 assessments identify 9 children with ASD and 23 assessments identify 8 children suggesting cost effectiveness analysis would be helpful in a larger study. Whilst we cannot accurately assess sensitivity in our study (as we have not assessed all the children in the sample for ASD), we used teacher based questionnaires (with ASD research diagnostic criteria) in 33 children with normal

For peer review only - http://bmjopen.bmj.com/site/about/guidelines.xhtml

V6 18. 06. 2020

EYFSP scores and low SCQ scores and none had more than two flagged areas of concern on the research diagnostic criteria symptom list for ASD (5-6 is the threshold for diagnosis). This suggests that further research may reveal an improved sensitivity when EYFSP is used as a pre-screen before SCQ.

This study has shown that there may be promising alternatives to existing assessment pathways for ASD (i.e. the use of EYFSP sub-score as a pre-screen tool, prior to SCQ screening). Advantages to the clinical process include the fact that information can be gathered from the school with those who know the child best (parents/carers and teacher) in one day in an environment known to the child, which may give a more accurate assessment. Previous studies using screening instruments with similar sample sizes have found a third of the sample are lost to follow up (11). Our study has vastly lower attrition because of the close link with the clinical teams into schools where parents are in regular contact. The early identification of ASD means that children can access the best educational placement early, and allows the local authority to plan its services and resources. It may resolve inequalities seen in previous studies where sections of the population do not come forward for assessment (17, 18).

This study identified a number of new children (n=9) with a diagnosis of ASD. This has enabled support to be established early. All of these children were from ethnic minorities suggesting that this process may be addressing inequalities in early diagnosis found in previous studies (17), although this would need further large scale research to confirm. In other studies using the Social Communication Questionnaire, when children score above the threshold but do not have ASD, approximately 90% have a neurodevelopmental disorder or developmental problem of some sort requiring identification and support (33). In our study (using the EYFSP) this was 100% with all children having identified support needs.

The study was limited by its size suggesting further larger district level research with cost-effectiveness analysis needs to take place.

For peer review only

Acknowledgements

Thanks to all the professionals who helped with the assessments including, Dr Claudia Salt, Dr Emily Williams, Natalie Langley, Halimah Hafiz, Nabihah Kauser, Ronnie Hartley, Prakash Thapa, Dr Alice Lambert. Thanks also to a range of people for their help to make this research possible including Professor John Wright, Dr David Sims, Rachael Vann, Sarah Oates, Amy Hart, Sophie Tully, Sarah Gates, Shelley Russell, Catarina Teige, Dani Varley, Dr Sue Lee and Rebecca Joy, Misbah Khan, Lydia Phillip.

A special thanks to Dr Stefan Williams and Dr Sujo Anathhanam who carried out earlier preparatory work prior to this study.

We would like to acknowledge our gratitude to the Bradford SHINE schools, Born in Bradford governors group, Connected Yorkshire Patient and Public Involvement panel, parents and their children and other stakeholders who have been involved in the study.

Contributor-ship statement:

Professor Barry Wright: Conceived of the presented idea, contributed to the design and delivery of the project and the writing up of the manuscript

Dr Konstantopoulou Kalliopi: Contributed to the design, delivery, data collection and the writing up of the manuscript

Kuldeep Sohal: Contributed to the design of the project and agreed with the manuscript's results and conclusions

Dr Brian Kelly: Contributed to the design of the project, completed the statistical analysis of the project and contributed to the writing of the manuscript

Dr Geoff Morgan: Contributed to the design, delivery of the project and agreed with the manuscript's results and conclusions

Cathy Hulin: Contributed to the design, overall organisation, data collection and writing up of the manuscript

Dr Sara Mansoor: Contributed to the design and delivery of the project

Professor Mark Mon-Williams: Contributed to the design of the project, agreed with the manuscript's results and conclusions

For peer review only - http://bmjopen.bmj.com/site/about/guidelines.xhtml

V6 18. 06. 2020

Competing interests: There are no competing interests for any author

Funding statement

The work was conducted within infrastructure provided by the Centre for Applied Education Research (www.caer.org.uk), and funded by the Department for Education through the Bradford Opportunity Area. The views expressed are those of the author(s), and not necessarily those of the NHS, the Bradford Local Authority or the Department for Education.

M. Mon-Williams was supported by a Fellowship from the Alan Turing Institute. The work was conducted within infrastructure provided by the Centre for Applied Education Research (funded by the Department for Education through the Bradford Opportunity Area) and ActEarly: a City Collaboratory approach to early promotion of good health and wellbeing funded by the Medical Research Council (grant reference MR/S037527/). Mon-Williams involvement was supported by the National Institute for Health Research Yorkshire and Humber ARC (reference: NIHR20016). The views expressed in this publication are those of the author(s) and not necessarily those of the National Institute for Health Research or the Departments of Health and Social Care or Education.

Data sharing statement:

Data are available upon reasonable request.

References

1. Baird G, Simonoff E, Pickles A, Chandler S, Loucas T, Meldrum D, et al. Prevalence of disorders of the autism spectrum in a population cohort of children in South Thames: the Special Needs and Autism Project (SNAP). The lancet. 2006;368(9531):210-5.
2. Szatmari P, Georgiades S, Bryson S, Zwaigenbaum L, Roberts W, Mahoney W, et al. Investigating the structure of the restricted, repetitive behaviours and interests domain of autism. Journal of Child Psychology and Psychiatry. 2006;47(6):582-90.
3. Georgiades S, Szatmari P, Zwaigenbaum L, Duku E, Bryson S, Roberts W, et al. Structure of the autism symptom phenotype: A proposed multidimensional model. Journal of the American Academy of Child & Adolescent Psychiatry. 2007;46(2):188-96.
4. Saggers B, Klug D, Harper-Hill K, Ashburner J, Costley D, Clark T, et al. Australian autism educational needs analysis–What are the needs of schools, parents and students on the autism spectrum2018.
5. French L, Kennedy EM. Annual Research Review: Early intervention for infants and young children with, or at-risk of, autism spectrum disorder: a systematic review. Journal of Child Psychology and psychiatry. 2018;59(4):444-56.
6. Postorino V, Sharp WG, McCracken CE, Bearss K, Burrell TL, Evans AN, et al. A systematic review and meta-analysis of parent training for disruptive behavior in children with autism spectrum disorder. Clinical Child and Family Psychology Review. 2017;20(4):391-402.
7. Rogge N, Janssen J. The Economic Costs of Autism Spectrum Disorder: A Literature Review. Journal of autism and developmental disorders. 2019:1-28.
8. Saccà A, Cavallini F, Cavallini MC. Parents of Children with Autism Spectrum Disorder: a systematic review. Journal of Clinical & Developmental Psychology. 2019;1(3).

For peer review only - http://bmjopen.bmj.com/site/about/guidelines.xhtml

9. Saunders B, Sim J, Kingstone T, Baker S, Waterfield J, Bartlam B, et al. Saturation in qualitative research: exploring its conceptualization and operationalization. Quality & quantity. 2018;52(4):1893-907.
10. Zwaigenbaum L, Bauman ML, Choueiri R, Fein D, Kasari C, Pierce K, et al. Early identification and interventions for autism spectrum disorder: executive summary. Pediatrics. 2015;136(Supplement 1):S1-S9.
11. Janvier YM, Harris JF, Coffield CN, Louis B, Xie M, Cidav Z, et al. Screening for autism spectrum disorder in underserved communities: Early childcare providers as reporters. Autism. 2016;20(3):364-73.
12. Towle PO, Patrick PA. Autism spectrum disorder screening instruments for very young children: a systematic review. Autism research and treatment. 2016;2016.
13. Crane L, Chester JW, Goddard L, Henry LA, Hill E. Experiences of autism diagnosis: A survey of over 1000 parents in the United Kingdom. Autism. 2016;20(2):153-62.
14. Commission CQ. Care Quality Commission: The State of Health Care and Adult Social Care in England-HC 838: The Stationery Office; 2013.
15. Agency UGST. Early Years Foundation Stage Profile 2018 [18th December 2019]. Available from: https://assets.publishing.service.gov.uk/government/uploads/system/uploads/attachment_data/file/790580/EYFSP_Handbook_2019.pdf.
16. Wright B, Mon-Williams M, Kelly B, Williams S, Sims D, Mushtaq F, et al. Investigating the association between early years foundation stage profile scores and subsequent diagnosis of an autism spectrum disorder: a retrospective study of linked healthcare and education data. BMJ Paediatrics Open. 2019;3(1).
17. Kelly B, Williams S, Collins S, Mushtaq F, Mon-Williams M, Wright B, et al. The association between socioeconomic status and autism diagnosis in the United Kingdom for children aged 5–8 years of age: Findings from the Born in Bradford cohort. Autism. 2019;23(1):131-40.
18. Nowell KP, Brewton CM, Allain E, Mire SS. The influence of demographic factors on the identification of autism spectrum disorder: A review and call for research. Review Journal of Autism and Developmental Disorders. 2015;2(3):300-9.
19. Harris B, McClain MB, Haverkamp CR, Cruz RA, Benallie KJ, Benney CM. School-based assessment of autism spectrum disorder among culturally and linguistically diverse children. Professional Psychology: Research and Practice. 2019;50(5):323.
20. Chestnut SR, Wei T, Barnard-Brak L, Richman DM. A meta-analysis of the social communication questionnaire: Screening for autism spectrum disorder. Autism. 2017;21(8):920-8.
21. Dickerson J, Bird PK, McEachan RR, Pickett KE, Waiblinger D, Uphoff E, et al. Born in Bradford's Better Start: an experimental birth cohort study to evaluate the impact of early life interventions. BMC Public Health. 2016;16(1):711.
22. Shire K, Andrews E, Barber S, Bruce A, Corkett J, Hill L, et al. Starting School: a large-scale start of school assessment within the ?Born in Bradford? longitudinal cohort [version 1; peer review: 1 approved, 1 approved with reservations]. Wellcome Open Research. 2020;5(47).
23. Wiggins LD, Bakeman R, Adamson LB, Robins DL. The utility of the Social Communication Questionnaire in screening for autism in children referred for early intervention. Focus on Autism and Other Developmental Disabilities. 2007;22(1):33-8.
24. Lee L-C, David AB, Rusyniak J, Landa R, Newschaffer CJ. Performance of the Social Communication Questionnaire in children receiving preschool special education services. Research in Autism Spectrum Disorders. 2007;1(2):126-38.
25. Schanding GT, Nowell KP, Goin-Kochel RP. Utility of the social communication questionnaire-current and social responsiveness scale as teacher-report screening tools for autism spectrum disorders. Journal of Autism and Developmental Disorders. 2012;42(8):1705-16.
26. Corsello C, Hus V, Pickles A, Risi S, Cook Jr EH, Leventhal BL, et al. Between a ROC and a hard place: decision making and making decisions about using the SCQ. Journal of Child Psychology and Psychiatry. 2007;48(9):932-40.
27. Barnard-Brak L, Brewer A, Chesnut S, Richman D, Schaeffer AM. The sensitivity and specificity of the social communication questionnaire for autism spectrum with respect to age. Autism research : official journal of the International Society for Autism Research. 2016 Aug;9(8):838-45. PubMed PMID: 26608837. Epub 2015/11/27. eng.

For peer review only - http://bmjopen.bmj.com/site/about/guidelines.xhtml

V6 18. 06. 2020

28. Rutter M, Le Couteur A, Lord C. Autism diagnostic interview-revised. Los Angeles, CA: Western Psychological Services. 2003;29:30.
29. Lord C, Rutter, M., DiLavore, P., Risi, S., Gotham, K., & Bishop, S. Autism diagnostic observation schedule-2nd edition (ADOS-2),. Western Psycological Corporation 2012. Los Angeles, CA:.
30. Organization WH. The ICD-10 classification of mental and behavioural disorders: Diagnostic criteria for research: World Health Organization; 1993.
31. Oosterling I, Rommelse N, de Jonge M, van der Gaag RJ, Swinkels S, Roos S, et al. How useful is the Social Communication Questionnaire in toddlers at risk of autism spectrum disorder? Journal of child psychology and psychiatry, and allied disciplines. 2010 Nov;51(11):1260-8. PubMed PMID: 20626528. Epub 2010/07/16. eng.
32. Surén P, Saasen-Havdahl A, Bresnahan M, Hirtz D, Hornig M, Lord C, et al. Sensitivity and specificity of early screening for autism. BJPsych open. 2019 May 17;5(3):e41. PubMed PMID: 31530312. Pubmed Central PMCID: PMC6537444. Epub 2019/09/19. eng.
33. Chandler S, Charman T, Baird G, Simonoff E, Loucas T, Meldrum D, et al. Validation of the social communication questionnaire in a population cohort of children with autism spectrum disorders. Journal of the American Academy of Child & Adolescent Psychiatry. 2007;46(10):1324-32.

For peer review only

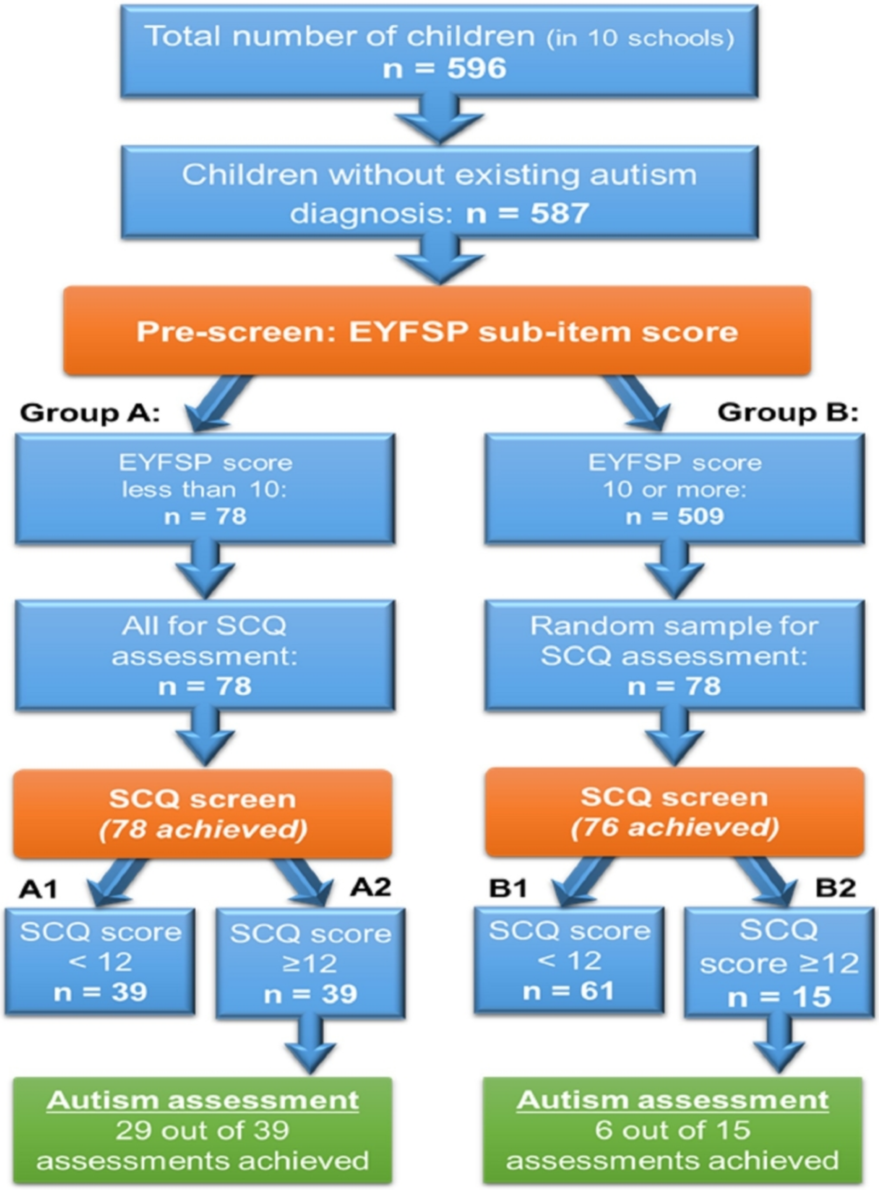

Figure 1

234x310mm (150 x 150 DPI)

STROBE Statement—Checklist of items that should be included in reports of ***cohort studies***

	Item No	Recommendation
**Title and abstract**	1	A systematic approach to school based assessments for Autism Spectrum Disorders to reduce inequalities? A feasibility study in ten primary schools.
		Objectives: This was a pilot study to explore whether the Early Years Foundation Stage Profile (EYFSP) carried out by teachers at the end of Reception year, followed by the Social Communication Questionnaire (SCQ) can lead to an earlier identification of children with Autism Spectrum Disorders (ASD), earlier access to intervention and reduce inequity in access to assessment and intervention.<br>Design: Pragmatic prospective cohort<br>Setting: 10 primary schools from the SHINE project in Bradford<br>Participants: 587 from 10 schools who transitioned from Reception to Year 1 in July 2017 and had the EYFSP completed were finally included in the study<br>Interventions: The assessment involved a team of three multidisciplinary staff who completed the Autism Diagnostic Interview Revised (ADI-R), the Autism Diagnostic Observation Schedule Version2 (ADOS-2), classroom observations with an ASD checklist, a teacher based ASD questionnaire and a final consensus meeting.<br>Primary outcome measure: NICE guideline compliant clinical diagnosis of ASD.<br>Secondary outcome measures: age of diagnosis, demographic data and feasibility parameters.<br>Results: Children who scored low on the EYFS were more likely to score above the SCQ threshold of 12(indicating potential autism), 50% compared to 19% of children not scoring low on the EYFS ($p < 0.001$). All children scoring above the SCQ received a full autism assessment; children who scored low on the EYFS were more likely to be diagnosed with autism (and other developmental issues) compared to those who did not score low on the EYFS.<br>Conclusions: We identified 9 new children with a diagnosis of ASD, all from ethnic minorities suggesting that this process may be addressing inequalities in early diagnosis found in previous studies. All children who scored above the threshold in the SCQ, required support and this was because the EYFSP questionnaire preceded it thereby including at risk children.
**Introduction**		
Background/rationale	2	Autism Spectrum Disorders (ASD) occur in approximately 1.6% of the UK population. Early identification and Early intervention has shown initial promise in improving outcomes. Whilst screening young children in early education settings has been attempted it identifies large numbers of children (14%) with relatively low numbers identified with ASD making cost effective whole population screening problematic. More nuanced approaches need to be developed. One promising approach would be to identify at risk populations and use screening and assessment processes within those groups. How to identify risk populations requires further research
Objectives	3	*Early Years Foundation Stage Profile (EYFSP)*<br>A large survey of parents in the UK describes late diagnosis in primary school despite symptoms being present from infancy and the Care Quality Commission found children with ASD having long waits for diagnosis and interventions. Recent studies suggest that using the Early Years Foundation Stage Profile may identify children with higher risk of having an ASD. The EYFSP is completed by teachers in England at the end of the reception year and scores 17 different domains of development in terms of whether a child is at an expected level, ahead or behind that level. It is used as a mechanism for flagging children who may need additional help in school and to benchmark UK school profiles.<br>*Equality of Access*<br>Recent work by the same group has also shown that the diagnosis of autism is less likely to be

For peer review only - http://bmjopen.bmj.com/site/about/guidelines.xhtml

		made early in families from poor backgrounds or from families from ethnic minority groups showing inequalities reported elsewhere. This problem with equity of access would be well served by having a more widely available process for identifying children for neurodevelopmental disorder assessment as early as is practicable. One mechanism for improving equity of access is school based assessment. *Reasons for feasibility work* To plan a larger study it is necessary to gather feasibility information for improved assessment processes. We report a feasibility study of a two stage screening process involving the EYFSP followed by an established well validated ASD screening questionnaire, the Social Communication Questionnaire (SCQ). We sought to test the feasibility of a process where children went through this screening process and were then assessed more comprehensively for ASD in schools with education and health professionals working together over one day.
**Methods**		
Study design	4	This research was set within the larger Born in Bradford cohort research. We obtained consent from 10 primary schools in an existing project, the SHINE project.
Setting	5	The assessments took place in those 10 schools in Bradford between September 2018 and July 2019. The assessment involved a team of three multidisciplinary staff drawn from a bank of child and adolescent mental health service (CAMHS) clinicians and educational psychologists. The assessment was completed in school in one day. One experienced clinician who was trained in the ADIR carried out this parent based semi-structured interview with a parent or primary care giver. Two other professionals (usually an educational psychologist and a clinical psychologist or child psychiatrist) trained in the ADOS-2 carried out this play/interaction based assessment with the child, using the most appropriate module depending on their developmental ability and language development. This was carried out by one person and observed by a second person and information shared during coding. One of the clinicians also observed the child in class with a bespoke ASD checklist. The clinicians went through a teacher based questionnaire related to the teacher's experiences of the child's skills and behaviour including the main symptoms of ASD using the World Health Organisation International Classification of Diseases Version 10 Research Diagnostic Criteria. Finally there was a consensus meeting with the three external assessors and the teacher identifying an overall consensus for the presence or absence of definite, possible or no difficulties in the 12 main research diagnostic criteria areas for Autism Spectrum Disorder diagnosis. In the afternoon each of the clinicians contributed to one single report using a range of sub-headings and organising material according to those sub-headings. This included a final consensus formulation, a description of strengths and difficulties and a range of recommendations.
Participants	6	596 children in year 5 were available in 10 primary schools and we approached all of those who had received an Early Years Foundation Stage Profile scored by their teachers at the end of reception year in the summer of 2017.
Variables	7	As agreed in ethical approvals the report fell short of making an NHS diagnosis (since this was a research project). Where appropriate it was suggested that referral was made through appropriate local assessment pathways with the report. A range of other recommendations were made including referral elsewhere such as speech and language therapy assessment, physical health checks or a proposed assessment for an Education Health Care Plan, educational psychology assessment or a range. Given the breadth of experience of the assessing professionals and the teacher, a number of possible recommendations for assessment were possible. *Feasibility Outcomes*

For peer review only

		Feasibility outcomes were collected such as numbers consenting, attrition rates after consent, acceptability of assessment elements, recording of any language or interpreting issues and the acceptability and completion of questionnaires.
Data sources/ measurement	8*	As described above
Bias	9	The assessments were performed by three independent clinicians using standardised tools according to the NICE guidelines criteria for an ASD assessment.
Study size	10	The teachers of children with low EYFSP scores and a 15% randomised sub-group of those with high scores (10 or more) completed a Social Communication Questionnaire (SCQ), which is a well-established validated autism screening questionnaire with good sensitivity and specificity scores. A threshold score of 12 or above on the SCQ was chosen, based on previous research; suggesting this is the best cut off for the optimum sensitivity to discriminate between children with and without ASD.
Quantitative variables	11	We carried qualitative interviews to obtain in-depth information from parents, teachers and clinicians about the acceptability, usefulness and real world provision of the assessment process.
Statistical methods	12	Data linkage allowed us to combine school and health data.<br>All those children and families with low EYFSP scores and above threshold SCQ (>12) were offered a NICE guideline compliant ASD Assessment, with additional clinical screening assessment for other developmental problems. A 15% randomised sub-group of those scoring high (10 or more) in EYFSP had the SCQ completed and those who scored high (10 or more) in EYFSP and 12 or above on the SCQ were then also assessed comprehensively in the same way. In order to check for false negatives we added an additional screening check where those children in the above groups given the SCQ scored below the threshold of 12 where their teacher filled in a narrative behaviour questionnaire mapping to the WHO research diagnostic criteria for ASD. This yields a score of 0-12 to identify areas of concern in any of the 12 symptom groups for ASD. Any child who had already had a diagnosis on the Autism Spectrum from the local diagnostic services was also noted.<br>Finally, sensitivity analysis was carried out using a cut of 15 or the SCQ instead of 12 as this has been used in some studies.
**Results**		
Participants	13*	There were 596 children in the 10 schools, 587 were included in the study as 9 children from this cohort had a pre-existing autism diagnosis. 14 families decided that they did not want to be part of the study and did not consent. Two families moved to a different school.
		Figures to be separately attached
Descriptive data	14*	There were 596 children in the 10 schools, 587 were included in the study as 9 children from this cohort had a pre-existing autism diagnosis (Figure 1). 14 families decided that they did not want to be part of the study and did not consent. Two families moved to a different school.<br>510 children scored 10 or above on the Early Years Foundation Stage Profile and 86 children scored 9 or below (at risk children). Of the 86 children scoring 9 or below, 8 (9%) of these children already had a diagnosis on the autism spectrum and the remainder were given the Social Communication Questionnaire (SCQ) with threshold results for 12 and 15 reported below.

For peer review only - http://bmjopen.bmj.com/site/about/guidelines.xhtml

SCQ Scores (those score 12 or above)

Autism Spectrum Disorder	Low EYFSP	Not Low EYFSP
Yes	9	0
No	20	6
Total	29	6

31% of those with Low EYSFP had diagnosis of ASD

SCQ Scores (those score 15 or above)

Autism Spectrum Disorder	Low EYFSP	Not Low EYFSP
Yes	8	0
No	13	3
Total	21	3

38% of those with Low EYSFP had diagnosis of ASD

All but one of the children who were met the criteria for a diagnosis of ASD had a SCQ of 15 or above.

Of the 510 children screened 10 or above (i.e. a low risk score) on the Early Years Foundation Stage Profile 1 child had a diagnosis on the Autism Spectrum already. We randomised 15% of these children to carry out the SCQ and so 78 families completed this with 15 of them scoring 12 or above on the SCQ with 61 scoring under 12 and 2 lost follow ups. The comprehensive Autism assessments described were offered to 54 children scoring greater than or equal to 12 on the SCQ from the children scoring 9 or below on the EYFSP with 39 carried out and with the random sub-group of those scoring 10 or above (n=15). Teachers to complete a comprehensive questionnaire based on the WHO research diagnostic criteria for ASD for 20 out of 39 children who scored 9 and below in EYFSP and less than 12 in SCQ as well as 33 out of 61 children who scored 10 or more in EYFSP and less than 12 in SCQ. We received a total of 53 questionnaires and none of them scored more than 2out of 12 on the research diagnostic criteria risk checklist, all below the level where a diagnosis of ASD would be likely. The large majority (88.68%) had 0 symptoms.

Outcome data	15*	See below
Main results	16	Those in group A (who score low on the EYFSP sub-score pre-screen) are more likely to be identified as potentially at risk of having ASD on the SCQ screening test compared to those in group B (those who do not score low on the EYFSP sub-score pre-screen); 50% of those in group A scored 12 or above on the SCQ, compared to 19% in group B (see table 1).

Table 1:

	SCQ Screen		
EYFSP sub-score pre-screen	High SCQ	Low SCQ	Total
Group A	50%	50%	78
Group B	19%	81%	78
Total	35%	65%	156

Pearson chi2(1) = 16.3137 p < 0.001

For peer review only - http://bmjopen.bmj.com/site/about/guidelines.xhtml

Group A are those scoring low on the EYFSP sub-score pre-screen score
Group B are those not scoring low on the EYFSP sub-score pre-screen score
High SCQ are those that score at least 12 on SCQ (potential autism)
Low SCQ are those that score less than 12 on SCQ (not potential autism)

Families of children who scored 12 or more on the SCQ screening tool who were then offered a full autism assessment, are described in table 2. Those who score low on the EYFSP sub-score pre-screen and then who go onto score high on the SCQ score (indicating potential autism) are much more likely to be diagnosed with ASD after the full assessment, compared to those in group B (those who did not score low on the EYFSP sub-score pre-screen and then who go onto score high on the SCQ score).

31% of those in group A with a SCQ of 12 or more met the research diagnostic criteria for ASD diagnosis.

None of those in group B with a SCQ of 12 or more met the research diagnostic criteria for ASD diagnosis.

Other analyses 17

Table 3 and 4 indicate the suggested referrals to other services that arose from the assessment, suggesting that this process may be useful in identifying children with a range of developmental problems and not simply those with ASD.

Table 3 Outcomes of assessments for those children with a SCQ score of 12 or above:

Referral to service	Group A2: Pre-screen: Low EYFSP sub-score (n = 29)	Group B2 Pre-screen: Not low EYFSP sub-score (n=6)	Groups A2 & B2 Total with autism assessment (n = 35)
Autism Spectrum Disorder	9 (31.0%)	0 (0%)	9 (25.7%)
Assessed Need for External (outside school system) support	22 (75.9%)	3 (50.0%)	25 (71.4%)
Assessed Need for Internal (within school system) support	29 (100%)	5 (83.3%)	34 (97.1%)
Assessed need for Internal or External Support	29 (100%)	6 (100%)	35 (100%)

Table 4: Recommendations from assessing clinicians about additional support needed for 35 assessed children

Group A: Group B Group A & B

For peer review only - http://bmjopen.bmj.com/site/about/guidelines.xhtml

For peer review only

Enacted Onward Referral to service	Pre-screen: Low EYFSP sub-score (n = 29)	Pre-screen: Not low EYFSP sub-score (n=6)	Total with autism assessment (n = 35)
Autism Spectrum Disorder	9 (31.0%)	0 (0%)	9 (25.7%)
Speech and Language Therapy Assessment	16 (55.2%)	3 (50.0%)	19 (54.3%)
Nurture Group/Encouragement of social interaction/monitoring	12 (41.4%)	4 (66.7%)	16 (45.7%)
Learning Needs Assessment	4 (13.8%)	2 (33.3%)	6 (17.1%)
In school Lego Based Therapy	3 (10.3%)	0 (0%)	3 (8.6%)
Parent Support	3 (10.3%)	0 (0%)	3 (8.6%)
Dyslexia Assessment	3 (10.3%)	0 (0%)	3 (8.6%)
Dyscalculia Assessment/Maths Skills Support	1 (3.4%)	0 (0%)	1 (2.9%)
Ed Psych/Cognitive Assessment	9 (31.0%)	0 (0%)	9 (25.7%)
Formal EHCP triggered	5 (17.2%)	0 (0%)	5 (14.3%)
Visual Aids and/or vision assessment	5 (17.2%)	0 (0%)	5 (14.3%)
In school Creative Activities groups	3 (10.3%)	0 (0%)	3 (8.6%)
Gross Motor Skills Support	3 (10.3%)	1 (16.7%)	4 (11.4%)
Physical Health Check	2 (6.9%)	0 (0%)	2 (5.7%)
In school Social Story intervention	2 (6.9%)	0 (0%)	2 (5.7%)
New Adaptations in Classrooms	6 (20.7%)	0 (0%)	6 (17.1%)
Occupational Therapy assessment	1 (3.4%)	0 (0%)	1 (2.9%)
Other group support	1 (3.4%)	0 (0%)	1 (2.9%)
Attention Concentration Support	6 (20.7%)	1 (16.7%)	7 (20.0%)

For peer review only

We checked the GP records of those 35 children identified as having low (29 children) and not low (6 children) EYFSP scores and 12 or above on the SCQ. Only 4 of these children had previously had any READ codes recorded for intellectual disability, language delay or disorder,

For peer review only - http://bmjopen.bmj.com/site/about/guidelines.xhtml

		ADHD or ASD, all four being recorded as having speech delay or disorder of speech and language. Two of these four children were assessed in our study as meeting the criteria for ASD. The remaining 31 children with low and not low EYFSP and SCQ > 12 had no GP recorded Read codes but all 31 had additional needs newly identified in our assessments (see table 4). This shows that of the 35 children 31 would gain new interventions as a result of our assessment processes that they were not currently accessing. All 9 of the children who were newly diagnosed with ASD by this research were from an ethnic minority background.
**Discussion**		
Key results	18	This study has shown that it is feasible to carry out a larger study of a new assessment care pathway for neurodevelopmental problems across a district.<br><br>In our trial the EYFSP pre-screen identified 13% of the pupil population (78 pupils scoring less than 10 on the EYFSP out of 587 pupils). Of this 13% of pupils half then go on to score high on the SCQ; so that approximately 6.5% of the pupil population would receive an autism assessment with the addition of the EYFSP pre-screen. This compares with 14% in similar early life screening studies without a pre-screen stage. This has potential cost-effectiveness benefits that we were unable to test but should be key parts of future research.<br>A recent paper suggests that, based on the cut off at 12, the sensitivity of the SCQ is 42% and the specificity 89%. Whilst we cannot accurately assess sensitivity in our study as we have not assessed all the children in the sample, we used teacher based questionnaires (with ASD research diagnostic criteria) in 33 children with normal EYFSP scores and low SCQ scores and none had more than 2 flagged areas of concern on the research diagnostic criteria symptom list for ASD (5-6 is the threshold for diagnosis). This suggests that further research may reveal an improved sensitivity when EYFSP is used as a pre-screen before SCQ.<br>This study has shown that there may be promising alternatives to existing assessment pathways for ASD (i.e. the use of EYFSP sub-score as a pre-screen tool, prior to SCQ screening). Advantages to the clinical process include the fact that information can be gathered from the school with those who know the child best (parents/carers and teacher) in one day in an environment known to the child, which may give a more accurate assessment. Previous studies using screening instruments with similar sample sizes have found a third of the sample are lost to follow up. Our study has vastly lower attrition because of the close link with the clinical teams into schools where parents are in regular contact. The early identification of ASD means that children can access the best educational placement early and allows the local authority to plan its services and resources. It may resolve inequalities seen in previous studies where sections of the population do not come forward for assessment.
Limitations	19	The study was limited by its size suggesting further larger district level research with cost-effectiveness analysis needs to take place.
Interpretation	20	This study identified a number of new children (n=9) with a diagnosis of ASD. This has enabled support to be established early. All of these children were from ethnic minorities suggesting that this process may be addressing inequalities in early diagnosis found in previous studies, although this would need further larger research to confirm. In other studies using the Social Communication Questionnaire, when children score above the threshold but do not have ASD, approximately 90% have a neurodevelopmental disorder or developmental problem of some sort requiring identification and support. In our study using the EYFSP this was 100% since all children had identified support needs.
Generalisability	21	The study gave promising results for a bigger study which could potentially include a larger

For peer review only

		number of participants
**Other information**		
Funding	22	The work was conducted within infrastructure provided by the Centre for Applied Education Research (www.caer.org.uk), and funded by the Department for Education through the Bradford Opportunity Area. The views expressed are those of the author(s), and not necessarily those of the NHS, the Bradford Local Authority or the Department for Education.

*Give information separately for exposed and unexposed groups.

Note: An Explanation and Elaboration article discusses each checklist item and gives methodological background and published examples of transparent reporting. The STROBE checklist is best used in conjunction with this article (freely available on the Web sites of PLoS Medicine at http://www.plosmedicine.org/, Annals of Internal Medicine at http://www.annals.org/, and Epidemiology at http://www.epidem.com/). Information on the STROBE Initiative is available at http://www.strobe-statement.org.

For peer review only

For peer review only - http://bmjopen.bmj.com/site/about/guidelines.xhtml

BMJ Open

A systematic approach to school based assessments for Autism Spectrum Disorders to reduce inequalities: A feasibility study in ten primary schools.

Journal:	*BMJ Open*
Manuscript ID	bmjopen-2020-041960.R2
Article Type:	Original research
Date Submitted by the Author:	08-Nov-2020
Complete List of Authors:	Wright, Barry; Hull York Medical School University of York<br>Konstantopoulou, Kalliopi; Leeds Community Healthcare NHS Trust, Child and Adolescent Psychiatry<br>Sohal, Kuldeep; Bradford Institute for Health Research<br>Kelly, Brian; Bradford Institute for Health Research<br>Morgan, Geoff; Educational Psychology Team, City of Bradford, Metropolitan District Council<br>Hulin, Cathy; Bradford Institute for Health Research<br>Mansoor, Sara; Bradford District Care NHS Foundation Trust<br>Mon-Williams, Mark; University of Leeds Department of Psychology
**Primary Subject Heading**:	Paediatrics
Secondary Subject Heading:	Mental health
Keywords:	Community child health < PAEDIATRICS, Child & adolescent psychiatry < PSYCHIATRY, Developmental neurology & neurodisability < PAEDIATRICS

SCHOLARONE™
Manuscripts

I, the Submitting Author has the right to grant and does grant on behalf of all authors of the Work (as defined in the below author licence), an exclusive licence and/or a non-exclusive licence for contributions from authors who are: i) UK Crown employees; ii) where BMJ has agreed a CC-BY licence shall apply, and/or iii) in accordance with the terms applicable for US Federal Government officers or employees acting as part of their official duties; on a worldwide, perpetual, irrevocable, royalty-free basis to BMJ Publishing Group Ltd ("BMJ") its licensees and where the relevant Journal is co-owned by BMJ to the co-owners of the Journal, to publish the Work in this journal and any other BMJ products and to exploit all rights, as set out in our licence.

The Submitting Author accepts and understands that any supply made under these terms is made by BMJ to the Submitting Author unless you are acting as an employee on behalf of your employer or a postgraduate student of an affiliated institution which is paying any applicable article publishing charge ("APC") for Open Access articles. Where the Submitting Author wishes to make the Work available on an Open Access basis (and intends to pay the relevant APC), the terms of reuse of such Open Access shall be governed by a Creative Commons licence – details of these licences and which Creative Commons licence will apply to this Work are set out in our licence referred to above.

Other than as permitted in any relevant BMJ Author's Self Archiving Policies, I confirm this Work has not been accepted for publication elsewhere, is not being considered for publication elsewhere and does not duplicate material already published. I confirm all authors consent to publication of this Work and authorise the granting of this licence.

For peer review only

V6 18. 06. 2020

A systematic approach to school based assessments for Autism Spectrum Disorders to reduce inequalities: A feasibility study in ten primary schools.

Professor Barry Wright[1]
Dr Konstantopoulou Kalliopi[2]
Kuldeep Sohal [3]
Dr Brian Kelly[3]
Dr Geoff Morgan [4]
Cathy Hulin[3]
Dr Sara Mansoor[5]
Professor Mark Mon-Williams[6]

1 Hull York Medical School, University of York

2 Leeds Community Healthcare NHS Foundation Trust

3 Bradford Institute for Health Research

4 Educational Psychology Team, City of Bradford, Metropolitan District Council

5 Bradford District Care NHS Foundation Trust

6 Department of Psychology, University of Leeds.

Corresponding author name and email address: Kalliopi Konstantopoulou

artekon84@gmail.com

For peer review only

V6 18. 06. 2020

Abstract:

Objectives: This was a pilot study to explore whether the Early Years Foundation Stage Profile (EYFSP) carried out by UK teachers within 'Reception' year, combined with the Social Communication Questionnaire (SCQ) can lead to an earlier identification of children with Autism Spectrum Disorders (ASD), earlier access to intervention, and reduce inequity in access to assessment and intervention.

Design: Pragmatic prospective cohort.

Setting: Ten primary schools from the SHINE project in Bradford.

Participants: Five hundred eighty seven (587) pupils from ten schools who transitioned from Reception to Year 1 in July 2017 and had the EYFSP completed were included in the final study.

Interventions: The assessment involved: a multidisciplinary team of three staff who completed the Autism Diagnostic Interview Revised (ADI-R), the Autism Diagnostic Observation Schedule Version2 (ADOS-2), classroom observations with an ASD checklist, a teacher based ASD questionnaire, and a final consensus meeting.

Primary outcome measure: NICE guideline compliant clinical diagnosis of ASD.

Secondary outcome measures: age of diagnosis, demographic data and feasibility parameters.

Results: Children with low scores on the EYFS were more likely to score above the SCQ threshold of 12 indicating potential autism (50% compared to 19% of children with high scores on the EYFS ($p < 0.001$)). All children scoring above SCQ threshold received a full autism assessment; children who scored low on the EYFS were more likely to be diagnosed with autism (and other developmental issues) compared to those who did not.

Conclusions: We identified nine new children with a diagnosis of ASD, all from ethnic minorities suggesting that this process may be addressing the inequalities in early diagnosis found in previous studies. All children who scored above the SCQ threshold required support (i.e. had a neurodevelopmental disorder), indicating the EYFSP questionnaire captured 'at risk' children.

For peer review only - http://bmjopen.bmj.com/site/about/guidelines.xhtml

V6 18. 06. 2020

Strengths and limitations of the study:

- Consent was sought from all parents regardless of language by flexible use of interpreters.
- Education and Health data was shared yielding significant benefits
- We conducted the SCQ (threshold of 12) with children who scored ≤ 9 in the EYFSP and a random sub-sample from the high EYFSP group (15% of children ≥ 10)
- All children with a score of ≥ 12 on the SCQ received a detailed comprehensive ASD assessment and the rest had a teachers' screening questionnaire
- Any child who had already had a diagnosis on the Autism Spectrum from the local diagnostic services was also noted

For peer review only

V6 18. 06. 2020

Introduction

What is Autism

Autism Spectrum Disorders (ASD) occur in approximately 1.6% of the UK population (1). ASD is a neurodevelopmental condition that often includes a range of repetitive behaviours, preoccupations and interests (2), and large developmental differences in social communication relative to neuro-typically developing individuals (3). ASD leads to a need for different approaches to education (4) and parenting (5), (6), which can be costly for local authorities (7) and stressful for parents and family (8); (9).

Early identification

Early identification and early intervention has shown promise in improving outcomes (10), (5). Screening young children in early education settings has been attempted, but captures relatively low numbers of children with ASD (11) despite large numbers (14%) being identified at risk. This has made cost effective whole population screening problematic (12), and there is a need for more nuanced approaches. The ability to use routine data to identify 'at risk' populations remains the holy grail of autism assessment (12). The need for such approaches was shown within a large survey of parents in the UK who reported receiving a diagnosis late in primary school despite symptoms being present from infancy (13). This was confirmed by the Care Quality Commission who reported that children with ASD experience long waits for diagnosis and interventions (14).

Early Years Foundation Stage Profile (EYFSP)

Recent studies suggest that using the Early Years Foundation Stage Profile (15) may identify children with higher risk of having an ASD (16). The EYFSP is completed by teachers in England at the end of the reception year and scores 17 different domains of development in terms of whether a child is at an expected level, ahead, or behind that level. It is used as a mechanism for flagging children who may need additional help in school and to benchmark UK school profiles (15).

For peer review only

V6 18. 06. 2020

Equality of Access

Recent work has shown that the diagnosis of autism is less likely to be made early in families from poor backgrounds or from families from ethnic minority groups (17) - reflecting inequalities reported elsewhere (18). This problem with equity of access could be addressed by having a more widely available process for identifying children with neurodevelopmental disorder as early as possible. One mechanism for improving equity of access is school based assessments (19).

Reasons for feasibility work

In order to plan a larger study, it is necessary to gather feasibility information for improved assessment processes. We report a feasibility study of a two stage screening process involving the EYFSP followed by an established well validated ASD screening questionnaire = the Social Communication Questionnaire (20). We sought to test the feasibility of a process where children went through this screening process and were then assessed more comprehensively for ASD *in schools* with education and health professionals working together over the course of one day.

Methodology

Background

The research was set within the larger Born in Bradford cohort study (21). We obtained consent from 10 primary schools in an existing consortium, the SHINE partnership. The SHINE group is a group of ten primary schools that act as a testbed for new approaches to improve services, reduce inequalities, and test innovations (22). We obtained ethical approval from University of Leeds and Bradford Teaching Hospitals NHS Foundation Trust (IRAS Number: 233328).

Consent

All parents were approached with a family information leaflet and a consent form. A researcher was available by phone, email, or face-to-face for those wishing to discuss the project further. Interpreters were available because many of the population had a first language that was not English.

For peer review only - http://bmjopen.bmj.com/site/about/guidelines.xhtml

V6 18. 06. 2020

Design

Five hundred and ninety six (596) children in Year 5 were available in 10 primary schools and we approached all of those who had received an Early Years Foundation Stage Profile scored by their teachers at the end of reception year in the summer of 2017.

The study was designed to test feasibility for a larger study.

Measures

A screening measure to identify children at risk was derived from five items of the EYFSP carried out by teachers at the end of reception year. The measure was taken from the four main symptom areas defined in the research diagnostic criteria for ASD – namely, social reciprocity, language and communication, imagination delays, and repetitive and stereotyped patterns of behaviour. This is described in more detail in a previous study (16). EYFSP assessment scores are recorded for children in Reception who are aged from 4 to 5 years. The assessments conducted by the clinicians occurred in Year 1 when children are typically aged 5 to 6 years of age. We chose a score threshold of 9 which a previous study found to be significantly (statistically) associated with over 50 times the risk of autism: 52.7 (95% CI: 25.2 - 110.5). (16). Children were dichotomously grouped into 'low' (≤ 9) and 'high' (≥ 10) scorers.

The teachers of children with low EYFSP scores and a 15% randomised sub-group of those with high scores (≥ 10) completed a Social Communication Questionnaire (SCQ) (23), which is a well-established validated autism screening questionnaire with good sensitivity and specificity scores. In previous studies the SCQ has been found to be helpful in identifying young children with ASD (24). A threshold score of ≥ 12 on the SCQ was chosen based on previous research (25), with claims that this is the best threshold with the optimum sensitivity to discriminate between children with and without ASD (26). A sensitivity analysis was prospectively agreed for the threshold of >=15

Methods

Data linkage allowed us to combine school and health data (26).

For peer review only - http://bmjopen.bmj.com/site/about/guidelines.xhtml

All those children and families with low EYFSP scores and above threshold SCQ (>12) were offered a NICE guideline compliant ASD Assessment, with additional clinical screening assessment for other neurodevelopmental problems including speech and language difficulties, learning difficulties, physical health problems, anxiety, and low self-esteem. A 15% randomised sub-group of those scoring high (≥ 10) in EYFSP had the SCQ completed and those who scored ≥ 10 in EYFSP and ≥ 12 on the SCQ were then also assessed comprehensively in the same way. There were 596 children in the 10 schools, 587 were included in the study as nine children from this cohort had a pre-existing autism diagnosis. Fourteen (14) families decided that they did not want to be part of the study and did not consent. Two families moved to a different school (Figure 1).

Insert Figure 1 here

In order to check for false negatives, we added an additional screening check for the children in the above groups. In cases where the SCQ was scored below the threshold of 12, teachers filled in a narrative behaviour questionnaire mapping to the WHO research diagnostic criteria for ASD (27). This yields a score of 0-12 to identify areas of concern in any of the twelve symptom groups for ASD (27). Any child who had already had a diagnosis on the Autism Spectrum from the local diagnostic services was also noted.

Finally, sensitivity analysis was carried out using a cut off 15 of the SCQ instead of 12 as this has been used in some studies (28).

Patient and Public Involvement

There has been strong involvement and co-design of this research through the Born in Bradford governors' group, the Connected Yorkshire Patient and Public Involvement panel, SHINE schools, parents, young people, and other stakeholders. They have been supportive in the preparatory workshops, feasibility phases and information design of the study. We consulted with the Connected Yorkshire Patient and Public Involvement panel throughout the life cycle of this study who acknowledged the importance to improve the pathway to earlier diagnosis of Child ASD to improve children's health and wellbeing outcomes. The panel consists of parents that have children diagnosed with Child ASD or have children that are on the neurodevelopmental disorder care pathways. Some of the discussions focussed

For peer review only - http://bmjopen.bmj.com/site/about/guidelines.xhtml

on the stigma within certain communities in Bradford with certain mental health issues which result in parents not acknowledging the child's health issues and seeking diagnosis earlier or seeking the appropriate support across health or the education sectors.

We have also extensively engaged with the Headteachers at the Bradford SHINE primary schools and other school staff who helped to inform parents of the study and in the recruitment phase. The Bradford SHINE schools were actively involved in the design and implementation phase and wish to acknowledge our gratitude in the supporting, co-designing, and active involvement in this study.

We disseminated information on the study via the local radio stations including Bradford Ramadan, BBC Radio 4 and via a following website to inform individuals of the research that is being undertaken in the region.

Website: https://caer.org.uk/autism-spectrum-conditions/

We have also disseminated the results of the study via dedicated workshops at the Born in Bradford event in September 2019 and a further workshop in January 2020. These workshops consisted of a broad range of professional stakeholders from health and education across the region that are involved in the care pathway as well as public representation. The discussions have evolved to how the research study could be scaled across the region.

The Autism Assessment

The assessments took place in the 10 schools in Bradford between September 2018 and July 2019. The assessment involved a team of three multidisciplinary staff drawn from a bank of child and adolescent mental health service (CAMHS) clinicians, and educational psychologists. The assessment was completed in school in one day. One experienced clinician who was trained in the ADI-R (27) carried out the parent based semi-structured interview with a parent or primary care giver. Two other professionals (usually an educational psychologist and a clinical psychologist or child psychiatrist) trained in the

For peer review only - http://bmjopen.bmj.com/site/about/guidelines.xhtml

ADOS-2 (29) carried out the play/interaction based assessment with the child, using the most appropriate module depending on the child's developmental ability and language development. The assessment was carried out by one person and observed by a second with information shared during coding. One of the clinicians also observed the child in class with a bespoke ASD checklist. The clinicians went through a teacher based questionnaire related to the teacher's experiences of the child's skills and behaviour, including the main symptoms of ASD, using the World Health Organisation International Classification of Diseases Version 10 Research Diagnostic Criteria (30). Finally there was a consensus meeting with the three external assessors and the teacher, identifying an overall consensus for the presence or absence of definite, possible or no difficulties in the twelve main research diagnostic criteria areas for Autism Spectrum Disorder diagnosis (28). In the afternoon, each of the clinicians contributed to one single report using a range of sub-headings, and organised material according to those sub-headings. This included a final consensus formulation, a description of strengths and difficulties and a range of recommendations. As agreed in ethical approvals the report fell short of making an NHS diagnosis (since this was a research project). It was suggested where appropriate that referral was made through appropriate local assessment pathways with the report. A range of other recommendations were made including referral elsewhere (e.g. speech and language therapy assessment), physical health checks or a proposed assessment for an Education Health Care Plan, educational psychology assessment or a range of actions. Given the breadth of experience of the assessing professionals and the teacher, a number of possible recommendations for assessment were possible.

Feasibility Outcomes

Feasibility outcomes were collected. These included numbers consenting, attrition rates after consent, acceptability of assessment elements, recording of any language or interpreting issues and the acceptability and completion of questionnaires.

We conducted qualitative interviews to obtain in-depth information from parents, teachers and clinicians about the acceptability, usefulness and real-world provision of the assessment process.

For peer review only - http://bmjopen.bmj.com/site/about/guidelines.xhtml

V6 18. 06. 2020

Results

Five hundred and ten (510) children scored ≥ 10 on the Early Years Foundation Stage Profile and 86 children scored ≤ 9 (at risk children). Of the 86 children scoring ≤ 9, eight (9%) already had a diagnosis on the autism spectrum and the remainder were given the Social Communication Questionnaire (SCQ) with threshold results for ≥ 12 and 15 reported below (31) (see table 1).

Table 1 – Percentage of children who met the threshold for ASD with threshold results ≥ 12 and 15 in the SCQ

**SCQ Scores (those score 12 or above)**		
**Autism Spectrum Disorder**	***Low EYFSP***	***Not Low EYFSP***
Yes	9	0
No	20	6
**Total**	**29**	**6**
31% of those with Low EYSFP had diagnosis of ASD		
**SCQ Scores** (those score 15 or above)		
**Autism Spectrum Disorder**	***Low EYFSP***	***Not Low EYFSP***
Yes	8	0
No	13	3
**Total**	**21**	**3**
38% of those with Low EYSFP had diagnosis of ASD		

All but one of the children who were met the criteria for a diagnosis of ASD had a SCQ of 15 or above meaning that 11 assessments were needed to identify one extra child with ASD.

Of the 510 children with ≥ 10 on the Early Years Foundation Stage Profile (i.e. a low risk score), one child already had a diagnosis on the Autism Spectrum. We conducted the SCQ on a randomised sample (15%) of these children. Seventy eight families completed the SCQ with fifteen scoring ≥ 12 on the SCQ, 61 scoring ≤ 11, and two lost during follow up. The comprehensive Autism assessments described were offered to 54 children scoring ≥ 12 on the SCQ from the children scoring 9 or below on the EYFSP with 39 carried out and with the random sub-group of those scoring 10 or above (n=15). Teachers completed a

For peer review only - http://bmjopen.bmj.com/site/about/guidelines.xhtml

V6 18. 06. 2020

comprehensive questionnaire based on the WHO research diagnostic criteria for ASD for 20 out of 39 children who scored ≤ 9 in EYFSP and ≤ 11 in SCQ, as well as 33 out of 61 children who scored ≥ 10 in EYFSP and ≤ 11 in SCQ. We received a total of 53 questionnaires and none of them scored more than 2 out of 12 on the research diagnostic criteria risk checklist, all below the level where a diagnosis of ASD would be likely. The large majority (88.68%) had zero indicators.

Those in group A (who scored low on the EYFSP sub-score pre-screen) were more likely to be identified as potentially at risk of having ASD on the SCQ screening test compared to those in group B (those who did not score low on the EYFSP sub-score pre-screen); 50% of those in group A scored ≥ 12 on the SCQ, compared to 19% in group B (see table 2).

Table 2: Comparison between EYFSP and SCQ groups

	SCQ Screen		
EYFSP sub-score pre-screen	**High SCQ**	**Low SCQ**	Total
Group A	50%	50%	78
Group B	19%	81%	78
**Total**	**35%**	**65%**	**156**

Pearson chi2(1) = 16.3137 $p < 0.001$

Group A are those scoring low on the EYFSP sub-score pre-screen score

Group B are those not scoring low on the EYFSP sub-score pre-screen score

High SCQ are those that score at least 12 on SCQ (potential autism)

Low SCQ are those that score less than 12 on SCQ (not potential autism)

Families of children who scored ≥ 12 on the SCQ screening tool who were then offered a full autism assessment, are described in Table 2. Those who scored low on the EYFSP sub-score pre-screen and then scored high on the SCQ score (indicating potential autism spectrum

For peer review only - http://bmjopen.bmj.com/site/about/guidelines.xhtml

disorder) were much more likely to be diagnosed with ASD after the full assessment, compared to those in group B (those who did not score low on the EYFSP sub-score pre-screen and then scored high on the SCQ score). Thirty one percent of those in group A with a SCQ of ≥ 12 met the research diagnostic criteria for ASD diagnosis. None of those in group B with a SCQ of ≥ 12 met the research diagnostic criteria for ASD diagnosis.

Table 3 and 4 indicate the suggested referrals to other services that arose from the assessment, indicating that this process may be useful in identifying children with a range of neurodevelopmental problems and not simply those with ASD.

Table 3 Assessment outcomes according to risk groups for children scoring at least 12 on the SCQ (potential autism):

	Group A2	Group B2	Groups A2 & B2
Referral to service	*Pre-screen: Low EYFSP sub-score (n = 29)*	*Pre-screen: Not low EYFSP sub-score (n=6)*	*Total with autism assessment (n = 35)*
**Autism Spectrum Disorder**	**9 (31.0%)**	**0 (0%)**	**9 (25.7%)**
**Assessed Need for External (outside school system) support**	**22 (75.9%)**	**3 (50.0%)**	**25 (71.4%)**
**Assessed Need for Internal (within school system) support**	**29 (100%)**	**5 (83.3%)**	**34 (97.1%)**
**Assessed need for Internal or External Support**	**29 (100%)**	**6 (100%)**	**35 (100%)**

Group A2 are those scoring low on the EYFSP sub-score pre-screen score and scoring at least 12 on SCQ (potential autism)

Group B2 are those not scoring low on the EYFSP sub-score pre-screen score and scoring at least 12 on SCQ (potential autism)

For peer review only - http://bmjopen.bmj.com/site/about/guidelines.xhtml

V6 18. 06. 2020

Table 4: Recommendations from assessing clinicians about additional support needed for 35 assessed children

Enacted Onward Referral to service	Group A2<br>Pre-screen: Low EYFSP sub-score (n = 29)	Group B2<br>Pre-screen: Not low EYFSP sub-score (n=6)	Group A2 & B2<br>Total with autism assessment (n = 35)
**Autism Spectrum Disorder**	9 (31.0%)	0 (0%)	9 (25.7%)
**Speech and Language Therapy Assessment**	16 (55.2%)	3 (50.0%)	19 (54.3%)
**Nurture Group/Encouragement of social interaction/monitoring**	12 (41.4%)	4 (66.7%)	16 (45.7%)
**Learning Needs Assessment**	4 (13.8%)	2 (33.3%)	6 (17.1%)
**In school Lego Based Therapy**	3 (10.3%)	0 (0%)	3 (8.6%)
**Parent Support**	3 (10.3%)	0 (0%)	3 (8.6%)
**Dyslexia Assessment**	3 (10.3%)	0 (0%)	3 (8.6%)
**Dyscalculia Assessment/Maths Skills Support**	1 (3.4%)	0 (0%)	1 (2.9%)
**Ed Psych/Cognitive Assessment**	9 (31.0%)	0 (0%)	9 (25.7%)
**Formal EHCP triggered**	5 (17.2%)	0 (0%)	5 (14.3%)
**Visual Aids and/or vision assessment**	5 (17.2%)	0 (0%)	5 (14.3%)
**In school Creative Activities groups**	3 (10.3%)	0 (0%)	3 (8.6%)
**Gross Motor Skills Support**	3 (10.3%)	1 (16.7%)	4 (11.4%)
**Physical Health Check**	2 (6.9%)	0 (0%)	2 (5.7%)
**In school Social Story intervention**	2 (6.9%)	0 (0%)	2 (5.7%)
**New Adaptations in Classrooms**	6 (20.7%)	0 (0%)	6 (17.1%)
**Occupational Therapy assessment**	1 (3.4%)	0 (0%)	1 (2.9%)
**Other group support**	1 (3.4%)	0 (0%)	1 (2.9%)
**Attention Concentration Support**	6 (20.7%)	1 (16.7%)	7 (20.0%)

Group A2 are those scoring low on the EYFSP sub-score pre-screen score, and scoring at least 12 on SCQ (potential autism)

Group B2 are those not scoring low on the EYFSP sub-score pre-screen score, and scoring at least 12 on SCQ (potential autism)

For peer review only - http://bmjopen.bmj.com/site/about/guidelines.xhtml

V6 18. 06. 2020

We checked the GP records of those 35 children identified as having low (29 children) and not low (6 children) EYFSP scores and ≥ 12 on the SCQ. Only four of these children had previously had any READ codes recorded for intellectual disability, language delay or disorder, ADHD or ASD, all four being recorded as having speech delay or disorder of speech and language. Two of these four children were assessed in our study as meeting the criteria for ASD. The remaining 31 children with low and not low EYFSP and SCQ > 12 had no GP recorded Read codes but all 31 had additional needs that were newly identified in our assessments (see table 4). This shows that of the thirty five children, 31 would gain new interventions as a result of our assessment processes that they were not currently accessing. All nine of the children who were newly diagnosed with ASD by this research were from an ethnic minority background. There were six boys and three girls that were diagnosed with ASD. From the six boys, there were three of Pakistani origin, two of Bangladeshi origin and one gypsy/traveller origin. From the three girls that were diagnosed with ASD, two are of Pakistani origin and one is of Bangladeshi heritage.

Qualitative findings

Associated qualitative research will be published separately. Feedback was requested from clinicians, school staff, assessed children's parents, and parents of children with a neurodevelopmental disorder from a patients' panel.

Both parents and clinicians were positive about school based assessment occurring (largely) in one day. This included the benefits of the child being in their normal routine and experiencing less anxiety than clinic visits. Parents were positive about not having to chase appointments and teachers were positive about involvement in all assessments.

Clinicians valued multidisciplinary working and the positives of access to rich school based data. A SENCO from one of the school mentioned that *"I liked that everybody can come together because you are in one place, everybody that knows the child is there and then it is kind of written as a team around the child…".* Parents commented that including school in the assessment process had helped teaching staff to adapt teaching and support for the child promptly. Challenges identified included difficulties coordinating different

For peer review only - http://bmjopen.bmj.com/site/about/guidelines.xhtml

professionals, children and parents together and last minute cancellations "*this process was highly dependent on administration both from the project and from school…*". Other themes highlighted related to the diagnosis and a range of responses relating to concern from a parent that their child's problems may be minimised or that they might be stigmatised.

Discussion

This study has shown that it is feasible to carry out a larger study of a new assessment care pathway for neurodevelopmental problems across a district. We found that schools were very willing to take part in the study, and showed great interest in early identification of children with autism, and other support needs. All schools we approached in Bradford agreed to take part and facilitate the study. Teachers were supportive, completing 53 of 55 questionnaires about the children who did not receive the full autism assessment. The acceptability to families is relatively good, although some families withdrew from the study and some had concerns about the consequences of their child receiving a diagnosis of ASD. This suggests that care needs to be taken when considering the emotional consequences for the family. It is good practice to provide parenting support to families of children newly diagnosed with ASD and this should be a key part of new assessment pathways or future research.

In our trial, the EYFSP pre-screen identified 13% of the pupil population (78 pupils scoring less than 10 on the EYFSP out of 587 pupils). From this population, half scored highly on the SCQ such that approximately 6.5% of the population received an autism identification with the addition of the EYFSP pre-screen. This compares with 14% (11) in similar early life screening studies without a pre-screen stage. This has potential cost-effective benefits that we were unable to test but should be key parts of future research.

A recent paper (32) suggests an SCQ threshold of 12, with a sensitivity of 42% and specificity 89%. Other authors have used 15 (31). Our analysis shows 35 assessments identify 9 children with ASD and 23 assessments identify 8 children suggesting cost effectiveness analysis would be helpful in a larger study. Whilst we cannot accurately assess sensitivity in our study (as we have not assessed all the children in the sample for ASD), we used teacher based questionnaires (with ASD research diagnostic criteria) in 33 children with normal

For peer review only - http://bmjopen.bmj.com/site/about/guidelines.xhtml

V6 18. 06. 2020

EYFSP scores and low SCQ scores and none had more than two flagged areas of concern on the research diagnostic criteria symptom list for ASD (5-6 is the threshold for diagnosis). This suggests that further research may reveal an improved sensitivity when EYFSP is used as a pre-screen before SCQ.

This study has shown that there may be promising alternatives to existing assessment pathways for ASD (i.e. the use of EYFSP sub-score as a pre-screen tool, prior to SCQ screening). Advantages to the clinical process include the fact that information can be gathered from the school with those who know the child best (parents/carers and teacher) in one day in an environment known to the child, which may give a more accurate assessment. Previous studies using screening instruments with similar sample sizes have found a third of the sample are lost to follow up (11). Our study has vastly lower attrition because of the close link with the clinical teams into schools where parents are in regular contact. The early identification of ASD means that children can access the best educational placement early, and allows the local authority to plan its services and resources. It may resolve inequalities seen in previous studies where sections of the population do not come forward for assessment (17, 18).

This study identified a number of new children (n=9) with a diagnosis of ASD. This has enabled support to be established early. All of these children were from ethnic minorities suggesting that this process may be addressing inequalities in early diagnosis found in previous studies (17), although this would need further large scale research to confirm. In other studies using the Social Communication Questionnaire, when children score above the threshold but do not have ASD, approximately 90% have a neurodevelopmental disorder or developmental problem of some sort requiring identification and support (33). In our study (using the EYFSP) this was 100% with all children having identified support needs.

The study was limited by its size suggesting further larger district level research with cost-effectiveness analysis needs to take place.

For peer review only - http://bmjopen.bmj.com/site/about/guidelines.xhtml

V6 18. 06. 2020

Acknowledgements

Thanks to all the professionals who helped with the assessments including, Dr Claudia Salt, Dr Emily Williams, Natalie Langley, Halimah Hafiz, Nabihah Kauser, Ronnie Hartley, Prakash Thapa, Dr Alice Lambert. Thanks also to a range of people for their help to make this research possible including Professor John Wright, Dr David Sims, Rachael Vann, Sarah Oates, Amy Hart, Sophie Tully, Sarah Gates, Shelley Russell, Catarina Teige, Dani Varley, Dr Sue Lee and Rebecca Joy, Misbah Khan, Lydia Phillip.

A special thanks to Dr Stefan Williams and Dr Sujo Anathhanam who carried out earlier preparatory work prior to this study.

We would like to acknowledge our gratitude to the Bradford SHINE schools, Born in Bradford governors group, Connected Yorkshire Patient and Public Involvement panel, parents and their children and other stakeholders who have been involved in the study.

Contributor-ship statement:

Professor Barry Wright: Conceived of the presented idea, contributed to the design and delivery of the project and the writing up of the manuscript

Dr Konstantopoulou Kalliopi: Contributed to the design, delivery, data collection and the writing up of the manuscript

Kuldeep Sohal: Contributed to the design of the project and agreed with the manuscript's results and conclusions

Dr Brian Kelly: Contributed to the design of the project, completed the statistical analysis of the project and contributed to the writing of the manuscript

Dr Geoff Morgan: Contributed to the design, delivery of the project and agreed with the manuscript's results and conclusions

Cathy Hulin: Contributed to the design, overall organisation, data collection and writing up of the manuscript

Dr Sara Mansoor: Contributed to the design and delivery of the project

Professor Mark Mon-Williams: Contributed to the design of the project, agreed with the manuscript's results and conclusions

For peer review only - http://bmjopen.bmj.com/site/about/guidelines.xhtml

V6 18. 06. 2020

Competing interests: There are no competing interests for any author

Funding statement

The work was conducted within infrastructure provided by the Centre for Applied Education Research (www.caer.org.uk), and funded by the Department for Education through the Bradford Opportunity Area. The views expressed are those of the author(s), and not necessarily those of the NHS, the Bradford Local Authority or the Department for Education.

M. Mon-Williams was supported by a Fellowship from the Alan Turing Institute. The work was conducted within infrastructure provided by the Centre for Applied Education Research (funded by the Department for Education through the Bradford Opportunity Area) and ActEarly: a City Collaboratory approach to early promotion of good health and wellbeing funded by the Medical Research Council (grant reference MR/S037527/). Mon-Williams involvement was supported by the National Institute for Health Research Yorkshire and Humber ARC (reference: NIHR20016). The views expressed in this publication are those of the author(s) and not necessarily those of the National Institute for Health Research or the Departments of Health and Social Care or Education.

Data sharing statement:

Data are available upon reasonable request.

References

1. Baird G, Simonoff E, Pickles A, Chandler S, Loucas T, Meldrum D, et al. Prevalence of disorders of the autism spectrum in a population cohort of children in South Thames: the Special Needs and Autism Project (SNAP). The lancet. 2006;368(9531):210-5.
2. Szatmari P, Georgiades S, Bryson S, Zwaigenbaum L, Roberts W, Mahoney W, et al. Investigating the structure of the restricted, repetitive behaviours and interests domain of autism. Journal of Child Psychology and Psychiatry. 2006;47(6):582-90.
3. Georgiades S, Szatmari P, Zwaigenbaum L, Duku E, Bryson S, Roberts W, et al. Structure of the autism symptom phenotype: A proposed multidimensional model. Journal of the American Academy of Child & Adolescent Psychiatry. 2007;46(2):188-96.
4. Saggers B, Klug D, Harper-Hill K, Ashburner J, Costley D, Clark T, et al. Australian autism educational needs analysis–What are the needs of schools, parents and students on the autism spectrum2018.
5. French L, Kennedy EM. Annual Research Review: Early intervention for infants and young children with, or at-risk of, autism spectrum disorder: a systematic review. Journal of Child Psychology and psychiatry. 2018;59(4):444-56.
6. Postorino V, Sharp WG, McCracken CE, Bearss K, Burrell TL, Evans AN, et al. A systematic review and meta-analysis of parent training for disruptive behavior in children with autism spectrum disorder. Clinical Child and Family Psychology Review. 2017;20(4):391-402.
7. Rogge N, Janssen J. The Economic Costs of Autism Spectrum Disorder: A Literature Review. Journal of autism and developmental disorders. 2019:1-28.
8. Saccà A, Cavallini F, Cavallini MC. Parents of Children with Autism Spectrum Disorder: a systematic review. Journal of Clinical & Developmental Psychology. 2019;1(3).

For peer review only - http://bmjopen.bmj.com/site/about/guidelines.xhtml

9. Saunders B, Sim J, Kingstone T, Baker S, Waterfield J, Bartlam B, et al. Saturation in qualitative research: exploring its conceptualization and operationalization. Quality & quantity. 2018;52(4):1893-907.
10. Zwaigenbaum L, Bauman ML, Choueiri R, Fein D, Kasari C, Pierce K, et al. Early identification and interventions for autism spectrum disorder: executive summary. Pediatrics. 2015;136(Supplement 1):S1-S9.
11. Janvier YM, Harris JF, Coffield CN, Louis B, Xie M, Cidav Z, et al. Screening for autism spectrum disorder in underserved communities: Early childcare providers as reporters. Autism. 2016;20(3):364-73.
12. Towle PO, Patrick PA. Autism spectrum disorder screening instruments for very young children: a systematic review. Autism research and treatment. 2016;2016.
13. Crane L, Chester JW, Goddard L, Henry LA, Hill E. Experiences of autism diagnosis: A survey of over 1000 parents in the United Kingdom. Autism. 2016;20(2):153-62.
14. Commission CQ. Care Quality Commission: The State of Health Care and Adult Social Care in England-HC 838: The Stationery Office; 2013.
15. Agency UGST. Early Years Foundation Stage Profile 2018 [18th December 2019]. Available from: https://assets.publishing.service.gov.uk/government/uploads/system/uploads/attachment_data/file/790580/EYFSP_Handbook_2019.pdf.
16. Wright B, Mon-Williams M, Kelly B, Williams S, Sims D, Mushtaq F, et al. Investigating the association between early years foundation stage profile scores and subsequent diagnosis of an autism spectrum disorder: a retrospective study of linked healthcare and education data. BMJ Paediatrics Open. 2019;3(1).
17. Kelly B, Williams S, Collins S, Mushtaq F, Mon-Williams M, Wright B, et al. The association between socioeconomic status and autism diagnosis in the United Kingdom for children aged 5–8 years of age: Findings from the Born in Bradford cohort. Autism. 2019;23(1):131-40.
18. Nowell KP, Brewton CM, Allain E, Mire SS. The influence of demographic factors on the identification of autism spectrum disorder: A review and call for research. Review Journal of Autism and Developmental Disorders. 2015;2(3):300-9.
19. Harris B, McClain MB, Haverkamp CR, Cruz RA, Benallie KJ, Benney CM. School-based assessment of autism spectrum disorder among culturally and linguistically diverse children. Professional Psychology: Research and Practice. 2019;50(5):323.
20. Chestnut SR, Wei T, Barnard-Brak L, Richman DM. A meta-analysis of the social communication questionnaire: Screening for autism spectrum disorder. Autism. 2017;21(8):920-8.
21. Dickerson J, Bird PK, McEachan RR, Pickett KE, Waiblinger D, Uphoff E, et al. Born in Bradford's Better Start: an experimental birth cohort study to evaluate the impact of early life interventions. BMC Public Health. 2016;16(1):711.
22. Shire K, Andrews E, Barber S, Bruce A, Corkett J, Hill L, et al. Starting School: a large-scale start of school assessment within the ?Born in Bradford? longitudinal cohort [version 1; peer review: 1 approved, 1 approved with reservations]. Wellcome Open Research. 2020;5(47).
23. Wiggins LD, Bakeman R, Adamson LB, Robins DL. The utility of the Social Communication Questionnaire in screening for autism in children referred for early intervention. Focus on Autism and Other Developmental Disabilities. 2007;22(1):33-8.
24. Lee L-C, David AB, Rusyniak J, Landa R, Newschaffer CJ. Performance of the Social Communication Questionnaire in children receiving preschool special education services. Research in Autism Spectrum Disorders. 2007;1(2):126-38.
25. Schanding GT, Nowell KP, Goin-Kochel RP. Utility of the social communication questionnaire-current and social responsiveness scale as teacher-report screening tools for autism spectrum disorders. Journal of Autism and Developmental Disorders. 2012;42(8):1705-16.
26. Corsello C, Hus V, Pickles A, Risi S, Cook Jr EH, Leventhal BL, et al. Between a ROC and a hard place: decision making and making decisions about using the SCQ. Journal of Child Psychology and Psychiatry. 2007;48(9):932-40.
27. Rutter M, Le Couteur A, Lord C. Autism diagnostic interview-revised. Los Angeles, CA: Western Psychological Services. 2003;29:30.
28. Barnard-Brak L, Brewer A, Chesnut S, Richman D, Schaeffer AM. The sensitivity and specificity of the social communication questionnaire for autism spectrum with respect to age.

For peer review only - http://bmjopen.bmj.com/site/about/guidelines.xhtml

V6 18. 06. 2020

Autism research : official journal of the International Society for Autism Research. 2016 Aug;9(8):838-45. PubMed PMID: 26608837. Epub 2015/11/27. eng.
29. Lord C, Rutter, M., DiLavore, P., Risi, S., Gotham, K., & Bishop, S. Autism diagnostic observation schedule-2nd edition (ADOS-2),. Western Psycological Corporation 2012. Los Angeles, CA:.
30. Organization WH. The ICD-10 classification of mental and behavioural disorders: Diagnostic criteria for research: World Health Organization; 1993.
31. Oosterling I, Rommelse N, de Jonge M, van der Gaag RJ, Swinkels S, Roos S, et al. How useful is the Social Communication Questionnaire in toddlers at risk of autism spectrum disorder? Journal of child psychology and psychiatry, and allied disciplines. 2010 Nov;51(11):1260-8. PubMed PMID: 20626528. Epub 2010/07/16. eng.
32. Surén P, Saasen-Havdahl A, Bresnahan M, Hirtz D, Hornig M, Lord C, et al. Sensitivity and specificity of early screening for autism. BJPsych open. 2019 May 17;5(3):e41. PubMed PMID: 31530312. Pubmed Central PMCID: PMC6537444. Epub 2019/09/19. eng.
33. Chandler S, Charman T, Baird G, Simonoff E, Loucas T, Meldrum D, et al. Validation of the social communication questionnaire in a population cohort of children with autism spectrum disorders. Journal of the American Academy of Child & Adolescent Psychiatry. 2007;46(10):1324-32.

Figure 1. Number of children who had an autism assessment according to the EYFSP and SCQ scores

For peer review only

For peer review only - http://bmjopen.bmj.com/site/about/guidelines.xhtml

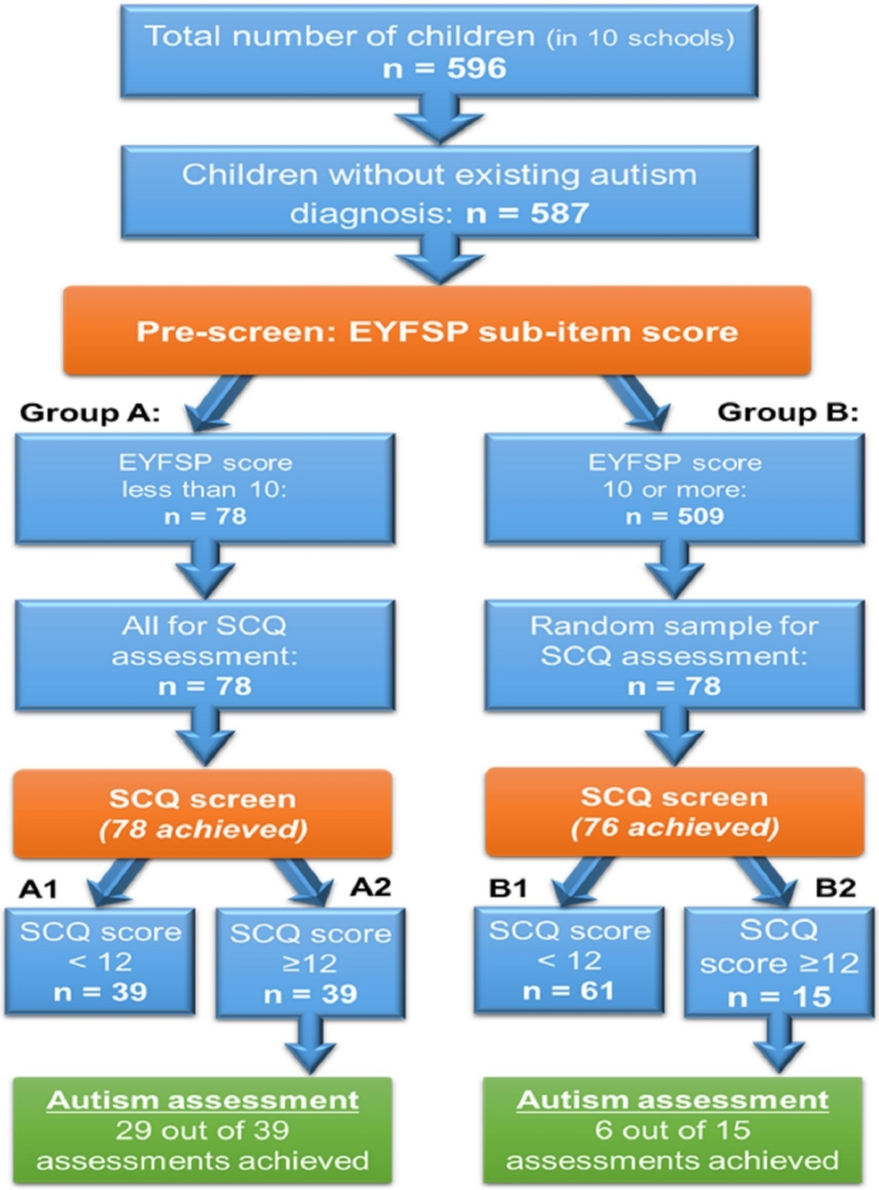

Figure 1

234x310mm (150 x 150 DPI)

STROBE Statement—Checklist of items that should be included in reports of ***cohort studies***

	Item No	Recommendation
**Title and abstract**	1	Page 1
		Page 2
**Introduction**		
Background/rationale	2	Page 4
Objectives	3	Page 4 - 5
**Methods**		
Study design	4	Page 5
Setting	5	Page 8 – 9
Participants	6	Page 6
Variables	7	Page 9
Data sources/ measurement	8*	Page 9
Bias	9	Page 7
Study size	10	Page 7
Quantitative variables	11	Page 7
Statistical methods	12	Page 6
**Results**		
Participants	13*	Page 10
		Figures to be separately attached
Descriptive data	14*	Page 10 - 11

For peer review only - http://bmjopen.bmj.com/site/about/guidelines.xhtml

For peer review only

Outcome data	15*	Page 11
Main results	16	Page 11 – 12
Other analyses	17	Page 13 - 14
**Discussion**		
Key results	18	Page 15
Limitations	19	Page 16.
Interpretation	20	Page 16
Generalisability	21	Page 16
**Other information**		
Funding	22	Page 18

*Give information separately for exposed and unexposed groups.

Note: An Explanation and Elaboration article discusses each checklist item and gives methodological background and published examples of transparent reporting. The STROBE checklist is best used in conjunction with this article (freely available on the Web sites of PLoS Medicine at http://www.plosmedicine.org/, Annals of Internal Medicine at http://www.annals.org/, and Epidemiology at http://www.epidem.com/). Information on the STROBE Initiative is available at http://www.strobe-statement.org.

For peer review only

For peer review only - http://bmjopen.bmj.com/site/about/guidelines.xhtml
